# Conformational aspects of polymorphs and phases of 2-propyl-1*H*-benzimidazole

**DOI:** 10.1107/S2052252518011685

**Published:** 2018-09-14

**Authors:** Fco. Javier Zuñiga, Aurora J. Cruz-Cabeza, Xabier M. Aretxabaleta, Noelia de la Pinta, Tomasz Breczewski, María Mar Quesada-Moreno, Juan Ramón Avilés-Moreno, Juan Jesús López-González, Rosa M. Claramunt, Jose Elguero

**Affiliations:** a Departamento Física Materia Condensada, Facultad de Ciencia y Tecnología, Universidad del País Vasco, Apartado 644, Bilbao E-48080, Spain; b School of Chemical Engineering and Analytical Sciences, The University of Manchester, The Mill, Sackville Street, Manchester M13 9PL, UK; c Departamento Física Aplicada II, Facultad de Ciencia y Tecnología, Universidad del País Vasco, Apartado 644, Bilbao E-48080, Spain; d Departamento de Química Física y Analítica, Universidad de Jaén, Campus Las Lagunillas, Jaén E-23071, Spain; e Departamento de Sistemas Físicos, Químicos y Naturales, Universidad Pablo de Olavide, Sevilla E-41704, Spain; f Departamento Química Orgánica y Bio-Orgánica, Facultad de Ciencias, Universidad Nacional de Educación a Distancia (UNED), Senda del Rey 9, Madrid E-28040, Spain; g Instituto de Química Médica, Centro de Química Orgánica Manuel Lora-Tamayo, CSIC, Juan de la Cierva 3, Madrid E-28006, Spain

**Keywords:** molecular crystals, polymorphism, phase transitions, conformational changes, energy minimization

## Abstract

This paper reports on the polymorphism of 2-propyl-1*H*-benzimidazole (2PrBzIm) induced by temperature change. Upon heating, an irreversible reconstructive-type phase transition at *T* = 384 K from the ordered form **I** (*P*2_1_2_1_2_1_) to a new polymorph, form **II**_HT_ (*Pcam*), was observed. The structural transformation between forms **I** and **II** involves significant changes in the crystal packing, as well as a key conformational variation around the propyl chain of the molecule. After the first irreversible phase transition, the **II**_HT_ form undergoes two further (reversible) phase transitions upon cooling at 361 K (**II**_RT_) and 181 K (**II**_LT_). All three phases (forms **II**_HT_, **II**_RT_ and **II**_LT_) have almost identical crystal packing and, given the reversibility of the conversions as a function of temperature, they are referred to as form **II** temperature phases. They differ, however, with respect to conformational variations around the propyl chain of 2PrBzIm. Energy calculations of the gas-phase conformational energy landscape of this compound about its flexible bonds allowed us to classify the observed conformational variations of all forms into changes and adjustments of conformers. This reveals that forms **I** and **II** are related by conformational change, and that two of the form **II** phases (HT and RT) are related by conformational adjustment, whilst the other two (RT and LT) are related by conformational change. We introduce the term ‘conformational phases’ for different crystal phases with almost identical packing but showing changes in conformation.

## Abstract 2

Temperature changes induced several solid-form conversions in 2-propyl-1*H*-benzimidazole. Three new crystal forms have been identified and characterized by various methods. A key structural aspect of these form conversions lies in the conformational changes around the propyl chains. Those changes drive polymorphic form changes, as well as low/high-temperature phase conversions. One phase conversion involves conformational change.

## 1. Introduction   

Benzimidazoles, indazoles and benzotriazoles are important organic molecules commonly found in biological systems. Besides their biological roles, these heterocyles have received considerable attention in materials science because they form crystalline materials able to exhibit notable conductivity and ferroelectric properties (Horiuchi *et al.*, 2010 ▸, 2012 ▸). The origin of these properties lies in the polar hydrogen-bonded chains present in their crystal structures and in the ability to easily transfer protons along those hydrogen bonds (Cosby *et al.*, 2015 ▸; Pulst *et al.*, 2016 ▸; Nagamani *et al.*, 2010 ▸). Some aspects related to the origin of the ferroelectricity can be understood in terms of the polarity exhibited by these compounds at the molecular and supramolecular levels. Imidazole rings exhibit large molecular dipole moments. In crystal structures, neighbouring imidazole rings linked by N—H⋯N hydrogen bonds form infinite polar chains resulting in very large net dipoles. Packing the polar chains in different crystal symmetries results in structures with ferroelectric or antiferroelectric properties. In these systems, structural phase transitions often occur that are associated with changes in temperature or pressure. The mechanisms of these transitions, at the molecular level, may involve proton transfer and/or conformational variations. Polymorphism is common for these compounds (Bernstein, 2002 ▸). For example, benzimidazole (Krawczyk & Gdaniec, 2005*a* ▸; Escande & Galigné, 1974 ▸), 2-ethyl-1*H*-benzimidazole (Cabildo *et al.*, 2015 ▸), omeprazole (Bhatt & Desiraju, 2007 ▸), 3-phenyl-1*H*-indazole (García *et al.*, 2002 ▸) or benzotriazole (Krawczyk & Gdaniec, 2005*b* ▸; Escande *et al.*, 1974 ▸) display polymorphism.

**Figure 1:**
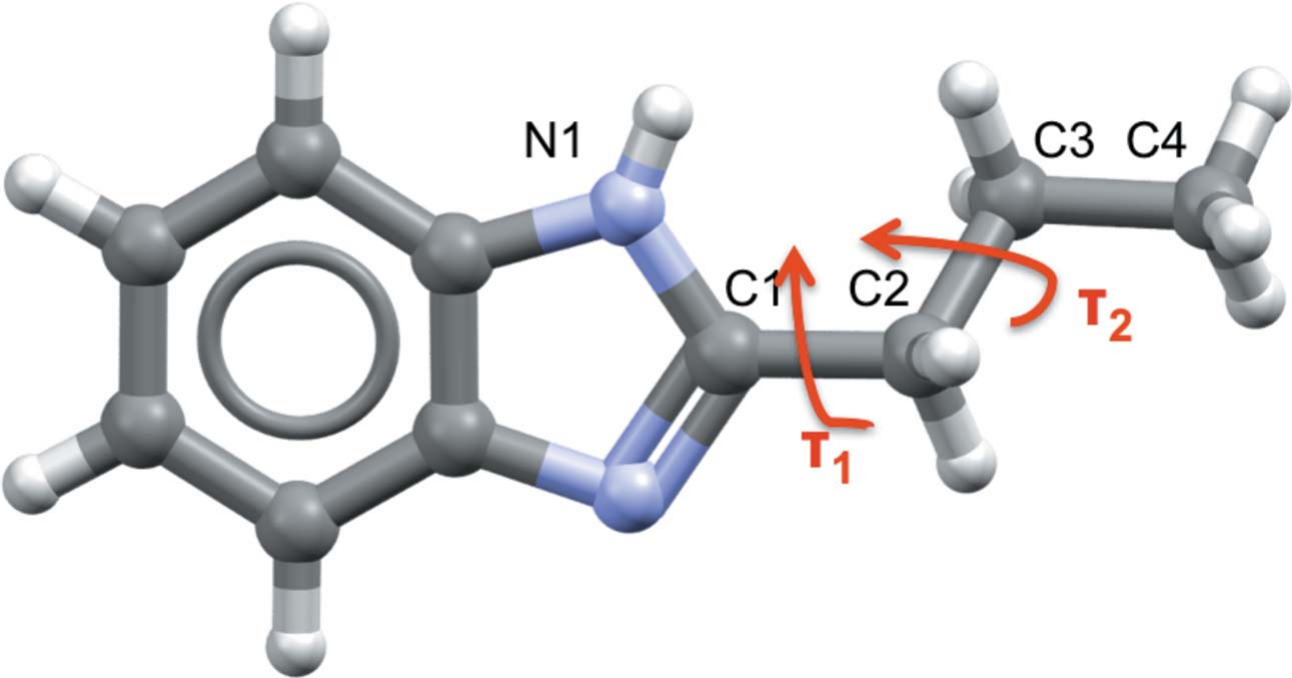
The molecular structure of 2PrBzIm; the torsion angles describing the conformation around the propyl chain are indicated.

In the case of 2-propyl-1*H*-benzimidazole (2PrBzIm, Fig. 1 ▸), the subject of this study, only one crystal structure, form **I**, has been reported to date. The structure of **I** attracted our attention because of its chirality (Quesada-Moreno *et al.*, 2017 ▸). We observed identical chiral responses, measured by vibrational circular dichroism (VCD), in form **I** 2PrBzIm samples prepared by different crystallization methods. This implies that identical enantiomeric excess is always produced independent of the crystallization method, an observation which we found remarkable, given that the enantiomeric purity of this compound arises only at the supramolecular level. The origin of this supramolecular chirality was rationalized (Quesada-Moreno *et al.*, 2017 ▸) even though the origin of the overall enantiomeric preference remains an open question.

**Figure 2:**
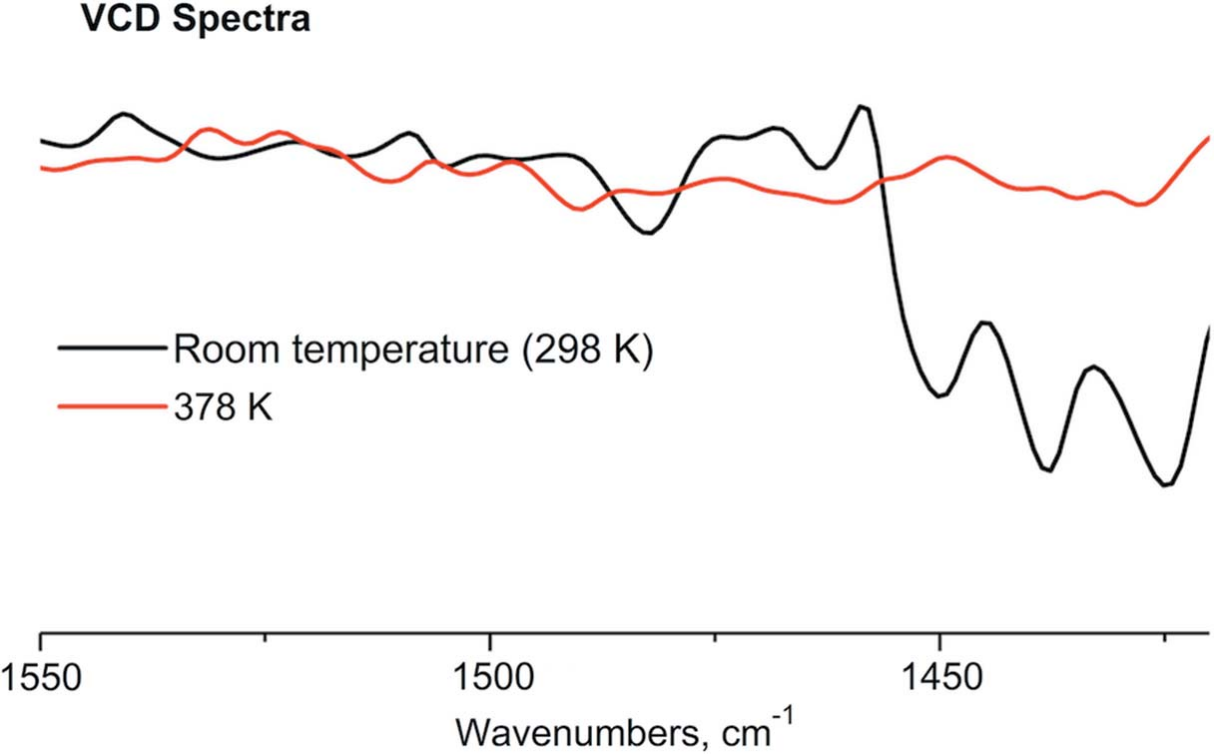
VCD spectra of 2PrBzIm crystals at room temperature in form **I** (black line) and after heating to 378 K in form **II** (red line).

Furthering our investigations on form **I** 2PrBzIm, we monitored the chiral-response changes of form **I** as a function of temperature. VCD recordings were obtained at different temperatures above 298 K. Once the samples were heated above ∼380 K, the VCD signals that were typical of form **I** disappeared completely (Fig. 2 ▸). We suspected this may be the result of a high-temperature solid–solid phase transition from a chiral to a new achiral form. We noticed that Ribeiro da Silva *et al.* (2004 ▸) had previously reported this transition, though no further details on it or the structure of the new form were published. These observations led us to investigate the polymorphism of 2PrBzIm further, which is the subject of this work.

## 2. Experimental   

### 2.1. Sample preparation   

Solid 2PrBzIm was purchased from Sigma–Aldrich. Samples of crystalline 2PrBzIm were taken directly from the commercial sample and prepared either by slow evaporation from a 3:1 Cl_2_CH_2_/hexane solution or by sublimation at 400 K on a hot stage. Further details of the experimental procedure used to grow crystals by sublimation are given in the supporting information (SI) (see Figs. S1 and S2).

### 2.2. Thermal analysis   

Differential scanning calorimetry (DSC) measurements were carried out on an MDSC Q-2000 calorimeter (TA Instruments) in the temperature range 120–450 K using heating and cooling rates of 10 K min^−1^ under a helium atmosphere. Polycrystalline samples of 2PrBzIm of mass ∼10–20 mg were hermetically sealed in aluminium pans. More details can be found in section S2 of the SI.

### 2.3. Vibrational circular dichroism   

VCD spectra of 2PrBzIm samples in fluoro­lube mulls (solid phase) were recorded using a JASCO FVS-4000 FTIR spectrometer, equipped with an MCT (2000–900 cm^−1^) detector. For the preparation of emulsions, a few milligrams of the crystal samples were mixed with fluoro­lube mineral oil in order to obtain suitable mulls (Conley, 1972 ▸; Sheppard, 2002 ▸). The mulls were measured in several positions by rotating the sample around both of the beam propagation axes (90 and 180°) and that perpendicular to them (180°), with the purpose of avoiding artefacts in the recorded VCD spectra. The recording resolution was 4 cm^−1^, with 8000 scans in blocks of 2000 (2000 scans for each orientation). For the baseline correction, the fluoro­lube signal was subtracted from the VCD spectra. IR and VCD recordings of 2PrBzIm samples were also recorded at various temperatures between room temperature and 378 K. Further details for the high-temperature measurement set-up are given in section S3 of the SI.

### 2.4. Scanning electron microscopy   

Scanning electron micrographs were recorded on a MERLIN Carl Zeiss instrument, working at voltages between 0.02 V and 30 kV. Prior to the measurements, the 2PrBzIm samples were metallized with gold.

### 2.5. Powder X-ray diffraction   

A powder sample of 2PrBzIm obtained by crystallization was prepared in a capillary and mounted on a Stoe STADI-P powder diffractometer in Debye geometry, with Ge-monochromated Cu *K*α_1_ radiation. Powder diffractograms were obtained in the temperature range 295–398 K in steps of 5 K. Temperature was monitored with an Oxford ITC controlling a heater device with a calibrated thermocouple near the capillary. The temperature stability during measurements was better than ±1 K. Experimental details and diffraction patterns can be found in the sections S4–S5 of the SI.

### 2.6. Single-crystal X-ray diffraction   

Single crystals suitable for XRD were prepared by sublimation. Good-quality crystals were selected and mounted on a goniometer and XRD diffraction data were collected on a SuperNova OD diffractometer using Cu *K*α radiation. Two sets of data were collected, one at 200 K and the second at 150 K. Structure solution was performed using *SIR2002* software (Burla *et al.*, 2003 ▸) and the refinement was carried out using the *JANA2006* package (Petříček *et al.*, 2006 ▸). Experimental refinement details are given in section S6 of the SI.

### 2.7. Computational methods   

#### 2.7.1. Molecular energy calculations   

The conformational energy of the 2PrBzIm molecule about torsion angles τ_1_ and τ_2_ (Fig. 1 ▸) was computed in the gas phase using density functional theory (DFT) methods as implemented in *GAUSSIAN16* (Frisch *et al.*, 2016 ▸). The B97D (Becke, 1997 ▸) hybrid functionals were used with a 6-311G** Pople basis set. This functional includes van der Waals dispersion corrections as derived by Grimme (2006 ▸). First, a potential energy surface (PES) scan about torsion angles τ_1_ and τ_2_ in the gas phase was computed for τ_1_ and τ_2_ from 0 to 180° and from 0 to 360°, respectively, in steps of 18°. Finally, geometries around the energy minima were fully optimized and the one-dimensional PES was then recomputed, freezing one of the torsion angles of the minimum-energy geometries.

#### 2.7.2. Relative lattice energy calculations   

The crystal structures of the 2PrBzIm polymorphs and phases were geometry-optimized using DFT-d periodic calculations, as implemented in the code *Vasp* (Version 5.4.1; Kresse & Hafner, 1993 ▸; Kresse & Furthmüller, 1996*a* ▸,*b* ▸). The PBE functional (Perdew *et al.*, 1996 ▸) was used together with PAW pseudopotentials (Blöchl, 1994 ▸; Kresse & Joubert, 1999 ▸) and Grimme’s van der Waals corrections (Grimme, 2006 ▸). Unit-cell volumes were optimized together with atomic positions using a kinetic energy cut-off of 520 eV for the plane waves. Structural relaxations were stopped when the calculated force on every atom was <0.005 eV Å^−1^, after which a single-point calculation was performed on the optimized structure for a final energy value. The Brillouin zone was sampled using the Monkhorst–Pack approximation on a grid of *k* points separated by approximately 0.04 Å^−1^. The resulting unit-cell electronic energies of the relaxed crystal structures were normalized by the number of molecules in the simulation cell. Energies are given relative to the form with the lowest electronic energy.

#### 2.7.3. Energy framework calculations   

Molecule–molecule interactions in the various polymorphs were calculated with the aid of *CrystalExplorer17* (Turner *et al.*, 2017 ▸) using the CE-B3LYP model for molecular energies (Turner *et al.*, 2014 ▸; Mackenzie *et al.*, 2017 ▸). Energy frameworks (Turner *et al.*, 2015 ▸) were visualized using an energy cut-off of 12 kJ mol^−1^.

## 3. Results and discussion   

### 3.1. Solid-form transformations induced by temperature changes   

**Figure 3:**
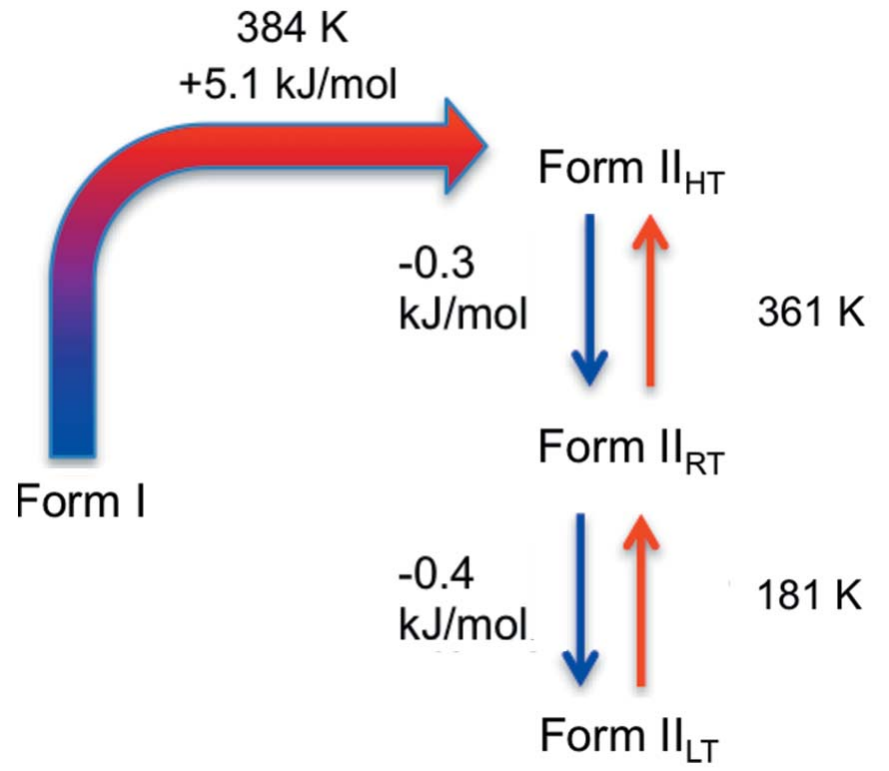
Conversions between polymorphs and phases of 2PrBzIm together with their transition temperatures and enthalpies, as observed in DSC.

Form **I** of 2PrBzIm, as crystallized, was investigated using DSC. First, the sample was cooled from room temperature to 120 K, with no observation of thermal events. After this first cooling cycle, the sample was heated from 120 K up to ∼410 K; during this heating cycle, a significant thermal event was observed at 384 K (DSC runs are shown in Fig. S3 of the SI). This thermal event corresponds to an irreversible solid–solid phase transition to a new form, which we refer to as **II**_HT_. The transition starts at around 370 K and completes just below 400 K with a maximum in *C*_p_ observed at 384 K and a transition enthalpy of +5.1±0.3 kJ mol^−1^ (an average of measurements using three samples; see Fig. S4). Although the new form melts at 431.6 K, 2PrBzIm exhibits significant sublimation from 400 K; thus, we did not heat the sample above 410 K. Finally, a cooling cycle from 410 K down to 120 K was performed. Here, we observed two consecutive (and reversible) phase transitions at 361 and 181 K with enthalpies (on cooling) of −0.3±0.1 and −0.4±0.2 kJ mol^−1^, respectively (see Figs. S5 and S6). These two transitions are much less drastic than the **I**–**II**_HT_ transition and are reversible. Given the similarity of these forms (which will be commented on in the sections below) and the full reversibility of the conversions with temperature, we chose to refer to these forms as different phases or temperature modulations of form **II** (Gavezzotti, 2007 ▸). Thus, form **II** exists in three different modulations at low temperature (LT, *T* < 181 K), room temperature (RT, *T* = 181–361 K) and high temperature (HT, *T* > 361 K). A summary of these transitions, polymorphs and modifications is given in Fig. 3 ▸.

It is important to highlight several observations in the thermal behaviour of this system. First, once form **I** converts to form **II**, there is no conversion back to **I**. Form **I** can only be obtained again through crystallization. Second, once form **II** is produced, **II**_HT_–**II**_RT_–**II**_LT_ conversions can be observed infinite times through consecutive cooling and heating cycles (Figs. S6 and S7). We observe, however, variations in the recorded enthalpies of the transitions upon changing the heating and cooling rates. This may be a consequence of the conversions being incomplete at faster rates; complete sample transformations are only accomplished at very low heating or cooling rates.

To further validate the phase transitions, variable-temperature powder X-ray diffraction (PXRD) was carried out starting from form **I** 2PrBzIm. Diffraction peaks characteristic of form **I** were observed at temperatures up to ∼380 K, above which, a drastic change in the PXRD pattern was observed, confirming that the first anomaly observed in DSC at 384 K corresponds to a reconstructive-type transition to a new form **II**_HT_. In the cooling cycle from 398 to 333 K, the diffraction pattern did not show any significant changes, which led us to conclude that the transformation between **II**_HT_ and **II**_RT_ does not involve major structural changes. PXRD patterns recorded in the temperature range 333–398 K in heating and cooling cycles and diagrams of forms **I** and **II** are given in the SI (see Figs. S9, S10 and S11).

Additionally, it was observed by VCD measurements how the chiral response of form **I** 2PrBzIm crystals changed as a function of temperature. Thus, Fig. S8 shows that the sample remains chiral after heating when the temperature does not reach 378 K. The signs of the VCD bands do not change and are only slightly red-shifted; decreased IR and VCD intensities are also noted. However, if the sample is heated above ∼380 K, the VCD signals belonging to form **I** disappear completely, because a solid–solid phase transition to the achiral **II**_HT_ form takes place, as discussed previously. The lack of chirality response is observed again if, later, the sample is cooled to room temperature after heating (form **II**_RT_).

### 3.2. Structure solution of form II and its temperature modifications   

Transformation of single crystals of form **I** to form **II**_HT_ (observed by DSC at 384 K) resulted in a polycrystalline sample of form **II**. Although these samples were analysed by PXRD and were indexed, production of single crystals of form **II** for XRD analysis by other means was required. This was achieved by growing form **II**_HT_ by sublimation. These single crystals remain intact across transitions between the temperature phases. A selected single crytal obtained by sublimation was analysed using XRD at 200 (modification **II**_RT_) and 150 K (modification **II**_LT_), and good-quality diffraction data were collected. This allowed structure solutions for the LT and RT modifications from XRD data. The diffraction data at high temperature (modification **II**_HT_), however, were of poor quality. As a consequence, we collected powder diffraction data at high temperatures instead (at 380 K) and used the form **II**_RT_ structure as a model on which then to carry out a Rietveld refinement on the high-temperature PXRD pattern. Crystal structure data for all modifications and details on their solution and refinement are given in Table 1 ▸ and in the SI, section S5 and Figs. S12–S14.

### 3.3. Conformational analysis of 2PrBzIm   

Prior to the analysis of the polymorphs and modifications, we performed some conformational analysis around τ_1_ and τ_2_ for 2PrBzIm in the gas phase. This analysis is essential in order to establish whether conformational variations that may occur between forms correspond to just conformational adjustments or conformational changes (Cruz-Cabeza & Bernstein, 2014 ▸). Two crystal conformations are related by adjustment if they both originate from the same gas-phase conformer (they remain in the same potential energy well). Two crystal conformations are related by conformational change if they originate from different gas-phase conformers (they belong to different basins of the potential energy well; Cruz-Cabeza & Bernstein, 2014 ▸).

**Figure 4:**
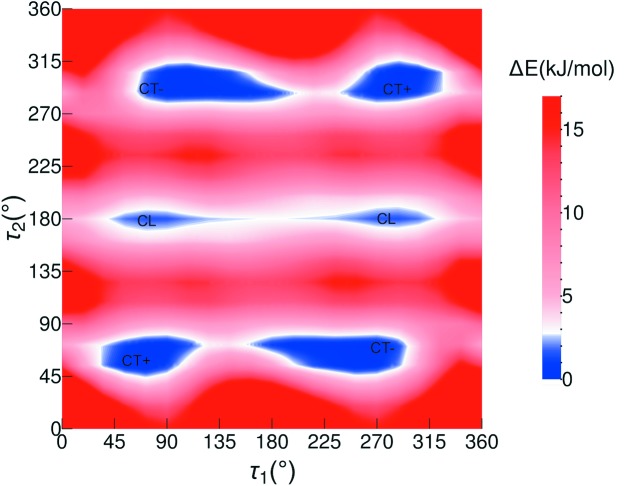
Conformational PES (B97d/6-311G**) of 2PrBzIm as a function of torsion angles τ_1_ and τ_2_. Positions of conformers CL, CT+ and CT− are labelled.

A PES scan at the B97D/6-311G** level of theory was performed as a function of angles τ_1_ and τ_2_ and is presented in Fig. 4 ▸. Another perspective view of the PES can be found in the SI (see Fig. S15). A relevant feature of the PES in Fig. 4 ▸ is the three long valleys for τ_2_ ≃ 63, 180 and 297°, with smooth hills along τ_1_. The energy surface is symmetrical for inversion through the central point (180, 180°), as well as sections τ_2_ ≃ 0 and 180° for τ_1_ ≃ ±180°. There are, in total, six minima in the PES of 2PrBzIm, of which three are symmetrically independent (Fig. 4 ▸), the other three being related by symmetry. The inverted minima have identical energies but inverted geometries (and torsion values). The exact coordinates of the minima on the PES are given in Table S2.

**Figure 5:**
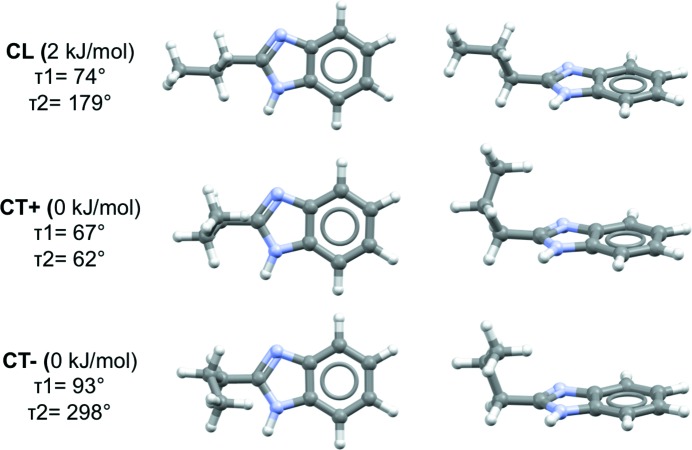
Relative conformational energies, geometries and visualization of the three symmetrically independent minima located in the PES (B97d/6-311G**).

The geometries of the three independent minima are illustrated in Fig. 5 ▸ (inverted versions of these have not been included). We note that τ_1_ for these minima lies around 67–103° (or 267–303° for the inverted conformers). We will refer to the PES conformers as CL (conformer linear) and CT (conformer twisted) which can be of type ‘+’ or type ‘−’. The main difference between the conformers thus lies around the τ_2_ torsion of the propyl chain. The CL conformer is in a local minimum (2 kJ mol^−1^) and is linear with torsion values of (78, 179°), (282, 181°) for the inverted version. Starting from the CL conformer, rotation of τ_2_ by ∼120° in one direction gives rise to a CT conformer, which we refer to as CT+, with torsion values of (72, 62°), (288, 298°) for the inverted CT+. Starting from the CL conformer, rotation of τ_2_ by 120° in the opposite direction results in conformer CT−, with torsion values of (103, 297°), (257, 63°) for the inverted version. We note that all our experimental polymorphs contain the identity as well as the reflected conformers, generated by mirror symmetry, with the exception of conformations in form **I**, which is a chiral space group. For simplicity, througout this paper, we will only refer to the three symmetrically independent minima.

**Figure 6:**
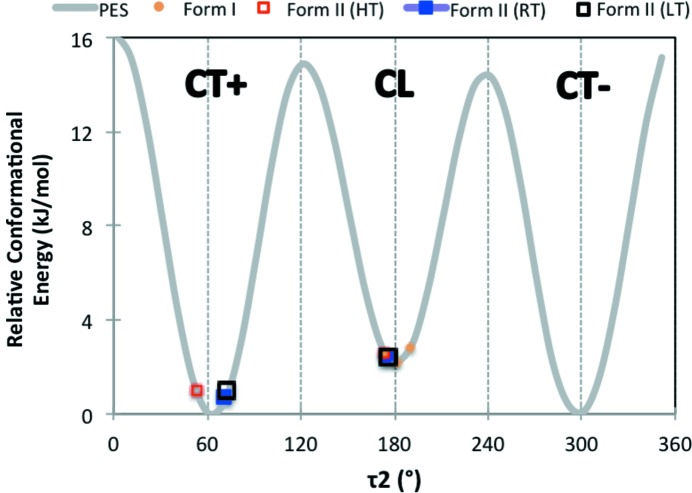
Conformational PES section (τ_1_ = 70°) along τ_2_.

CT+ and CT− are both quasi-isoenergetic (see Table S2) and are the most stable conformers, whilst CL is a local conformer. The positions of these conformers on the PES are shown in Fig. 4 ▸. Conversion between the two twisted conformers, CT+ and CT−, requires a 180° rotation of τ_1_ and the crossing of a small energy barrier of ∼4.5 kJ mol^−1^. Conversion of the linear conformer CL to a twisted conformer (CT+ or the CT−), however, requires a 120° rotation of τ_2_ and the crossing of a larger energy barrier of ∼14 kJ mol^−1^. The energy barrier of the CL-to-CT conversion is typical of alkane rotations. As we will discuss later, the CL-to-CT conversion is the conformational transition observed experimentally; thus, the relevant section of the PES for this transition is given in Fig. 6 ▸, together with the observed experimental conformations in the various crystal structures.

### 3.4. Analysis of forms and transformations   

#### 3.4.1. Polymorphs, modifications and conformations   

The crystal structure of form **I** 2PrBzIm [Cambridge Structural Database (CSD; Groom *et al.*, 2016 ▸) refcode OHUZUO] has been described elsewhere (Cabildo *et al.*, 2015 ▸; Quesada-Moreno *et al.*, 2017 ▸). Form **I** is orthorhombic (*P*2_1_2_1_2_1_) and crystallizes with four independent molecules in the asymmetric unit in a pseudo-tetragonal cell. Form **II** modifications at HT and RT have orthorhombic *Pcam* and *Pca*2_1_ symmetries, respectively, with two independent molecules in the asymmetric units. In the HT form, the molecules are disordered between two equally populated positions that are symmetry related by the mirror plane *m*. The LT modification involves a loss of symmetry (together with a doubling of a unit-cell length) and has four independent molecules in the asymmetric unit. The nature of this LT transition is typical of low-temperature phases.

In terms of stability, form **I** (which is often obtained by crystallization) is the most stable form at low temperatures since the **I**–**II** transition is endothermic. This is confirmed by our lattice-energy calculations. The form **II** modifications are between 4–6 kJ mol^−1^ higher in lattice enthalpy than form **I,** as measured by DSC and computed with DFT-d. The DSC measurements and the lattice-energy calculations are in excellent agreement. We notice that the crystal structures of forms **II**_HT_ and **II**_RT_ optimize to the same potential energy minimum. As expected, form **II** modifications have the following order of lattice enthalpies: Δ*H*(**II**_LT_) > Δ*H*(**II**_RT_) > Δ*H*(**II**_HT_). In these transitions, as the temperature is increased, a loss of lattice enthalpy is compensated for by an increase in lattice entropy. We note that our system follows the density rule (Burger & Ramberger, 1979 ▸).

Conformational descriptors of the four forms studied here are summarized in Table 2 ▸ (and details of torsion angles are available in Table S1). We only observe either the CL or the CT+ conformer in the different forms; the CT− conformer is never observed. Form **I** consists of adjustments of the linear conformer (CL). In the **I**–**II**_HT_ transition, half of the symmetry-independent molecules go through a conformational change from CL to CT+. Upon cooling, in the **II**_HT_–**II**_RT_ transition, we only observe conformational adjustments. On further cooling, in the **II**_RT_–**II**_LT_ transition, half of the remaining molecules in CL conformations undergo a conformational change to CT+.

We note that, on average, adjustments around τ_1_ are more significant than those around τ_2_. For example, the τ_1_ minimum lies around 70°, but it is observed in various forms in the ranges 40–50° (CL conformations) and 44–84° (CT+ conformations). These correspond to adjustment values of τ_1_ of around 14–26°. By contrast, the τ_2_ minimum lies around 180 and 60°, but is observed in various forms in the ranges 168–177 (CL conformations) and 67–85° (CT+ conformations). These correspond to adjustment values of τ_2_ of around 2–11 (CL conformations) and 9–18° (CT+ conformations).

#### 3.4.2. Type A and B hydrogen bonding and layers   

**Figure 7:**
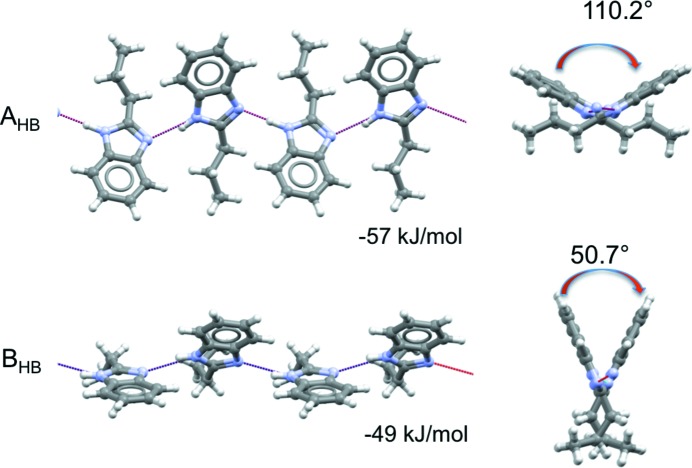
Type A (top) and type B (bottom) hydrogen-bonded chains in 2PrBzm. Perpendicular (left) and parallel (right) views of the hydrogen-bonded chain direction.

In forms **I** and **II**_HT_ of 2PrBzIm, two distinct types of hydrogen-bonded chain motifs are found. Both chain motifs are generated by local glide-plane symmetry (Fig. 7 ▸), their main difference being the angle between the aromatic rings of interacting 2PrBzIm molecules in the chain. If we view the chains along their hydrogen-bonded axis (Fig. 7 ▸, right), we can appreciate that the angle between the rings in type A chains is 110°, whilst that in type B is much smaller (51°). A transformation from a type A (extended configuration) to a type B hydrogen-bonded chain (folded configuration) requires a cooperative ‘butterfly-type’ movement, in which the ring–ring angle closes from 110 to 51°, together with a conformational change from a CL to a CT+ conformation. The hydrogen-bond energies in the two chains are −57 and −49 kJ mol^−1^ for types A and B, respectively, as calculated with *CrystalExplorer17* (see Section 2.7.3). These two types of hydrogen-bonded chains then pack in very different ways, forming type A and B layers.

**Figure 8:**
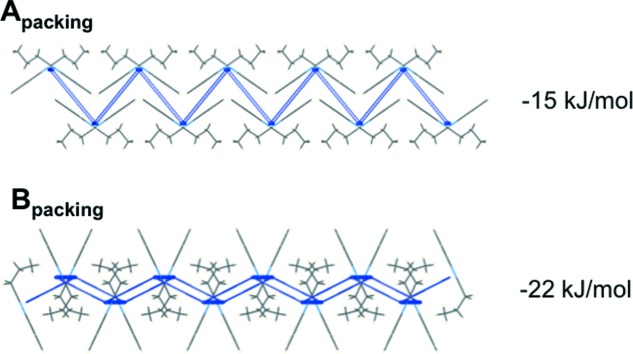
Interactions between type A (top) and type B (bottom) hydrogen-bonded chains in 2PrBzIm.

Type A chains pack through aromatic interactions between adjacent chains, as shown in Fig. 7 ▸. The rings of 2PrBzm molecules in adjacent chains interact with each other through aromatic stacking (middle of the A layer) whilst the alkyl chains are exposed at the edges of the layers. This results in a type-A layer of a hydro­phobic nature. Type B chains pack through ring-to-alkyl interactions between adjacent chains, as shown in Fig. 8 ▸. The rings of 2PrBzIm molecules in one chain interact through van der Waals forces with the alkyl groups of 2PrBzIm in adjacent chains. We note that the alkyl chains in type B layers alternate with the rings. Type B layers expose the aromatic rings at the edges of the layers. Interactions of the packing between adjacent layers were calculated to be −15 kJ mol^−1^ for type A and −22 kJ mol^−1^ for type B.

#### 3.4.3. Forms **I** and **II**_HT_   

**Figure 9:**
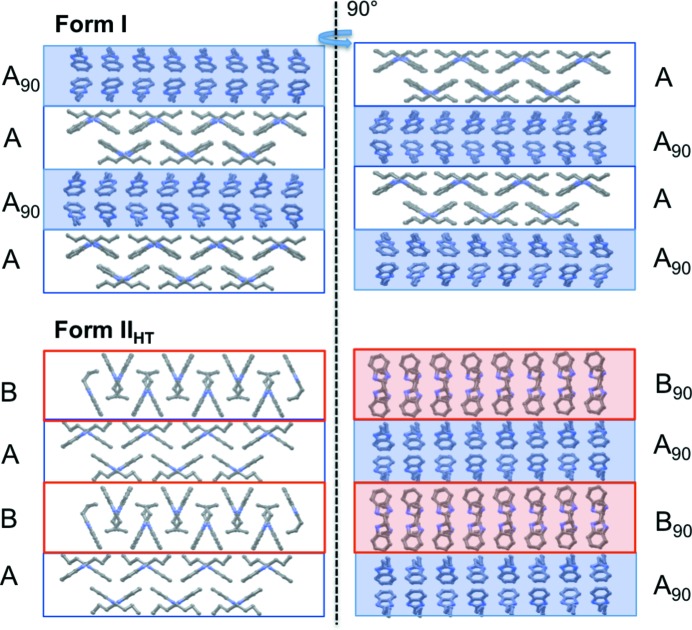
Illustration of the structural similarities and differences between form **I** (top) and one of the disordered configurations in **II**_HT_ (bottom). Type A layers are blue and type B layers are red. Form **I** (top): view along the *a* axis (left) and the *b* axis (right). Form **II**_HT_ (bottom): view along the *c* axis (left) and the *a* axis (right).

**Figure 10:**
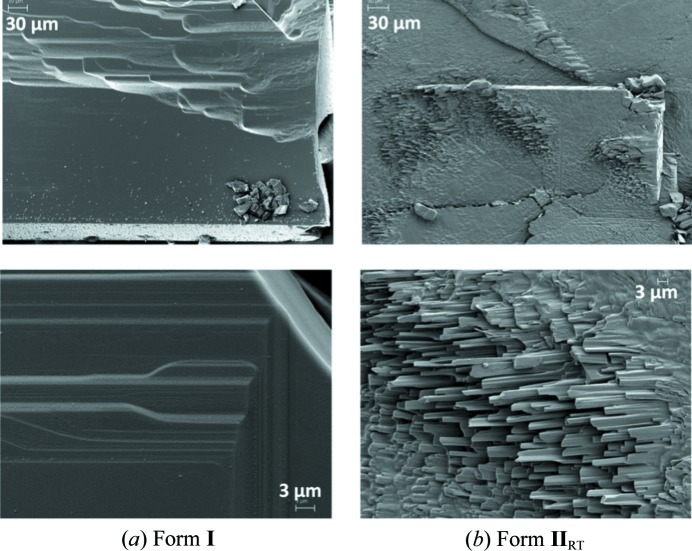
SEM images of the microstructures of form **I** and form **II**_RT_ crystals.

Forms **I** and **II**_HT_ can be described with the aid of type A and B layers, and this is illustrated in Fig. 9 ▸; the hydrogen atoms have been removed to aid visualization. Form **I** can be understood as a result of stacking type A layers alone. In form **I**, consecutive A layers are rotated by 90° with respect to each other. This results in the hydrogen-bonded chains being perpendicular to each other in consecutive layers. The packing of form **I** can be understood as an A–A_90_–A–A_90_ type. Half of the hydrogen-bonded chains are aligned with the *a* axis (Fig. 9 ▸, upper left), whereas the other half are aligned with the *b* axis (Fig. 9 ▸, upper right). In form **I**, the *a* and *b* axes are equivalent. This structure results in two strong and equivalent crystal-growth directions along the hydrogen-bonded chains; as a consequence, form **I** crystallizes as thin plates with clearly dominant (001) faces. SEM images (Fig. 10 ▸) of form **I** plates are illustrative and show steps along the (001) direction. These steps must be a consequence of layer-by-layer growth.

Considering one of the two disordered molecular configurations, form **II**_HT_ arises from a mixed packing of A- and B-type layers. The packing of form **II**_HT_ can be understood as A–B–A–B type. Contrary to form **I**, all hydrogen-bonded chains are aligned along the same direction (the *c* axis). Form **II**_HT_ has a single, very strong crystal-growth direction along the hydrogen-bonded chains (*c* axis), which is clearly manifested macroscopically in the form of needle-like morphologies. What is more, all hydrogen-bonded chains are aligned and point in the same direction. This results in a polar structure along the *c* axis (needle axis) in which the face at one end of the needle is rich in hydrogen-bond donors and the other end is rich in hydrogen-bond acceptors. The polar nature of one of the two disordered configurations of the structure results in polar growth, illustrated in the SEM images in Fig. 9 ▸, where images of form **II**_HT_, obtained by heating form **I**, show that form **II** needles only grow in one direction.

#### 3.4.4. Transformation between forms **I**, **II**_HT_, **II**_RT_ and **II**_LT_   

**Figure 11:**
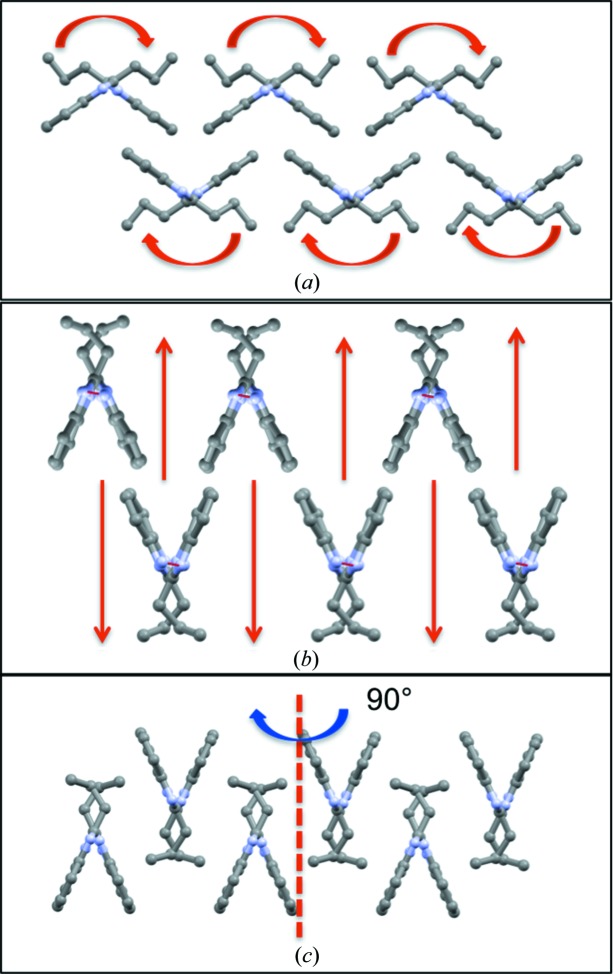
Steps required to convert an A_90_ layer into a B layer.

Structurally, for form **I** (A–A_90_–A–A_90_) to convert to form **II** (A–B–A–B), every other A layer needs to convert to B and rotate by 90°. The necessary changes include (Fig. 11 ▸): (*a*) ‘butterfly closing’ of the hydrogen-bonded chain, coupled with a CL→CT+ conformational change to convert the chains from type A to type B, (*b*) vertical shifting of those chains and (*c*) rotation of the entire layer by 90°. This set of steps aids the understanding of the structural changes and is, by no means, a description of the mechanism of the transformation.

**Figure 12:**
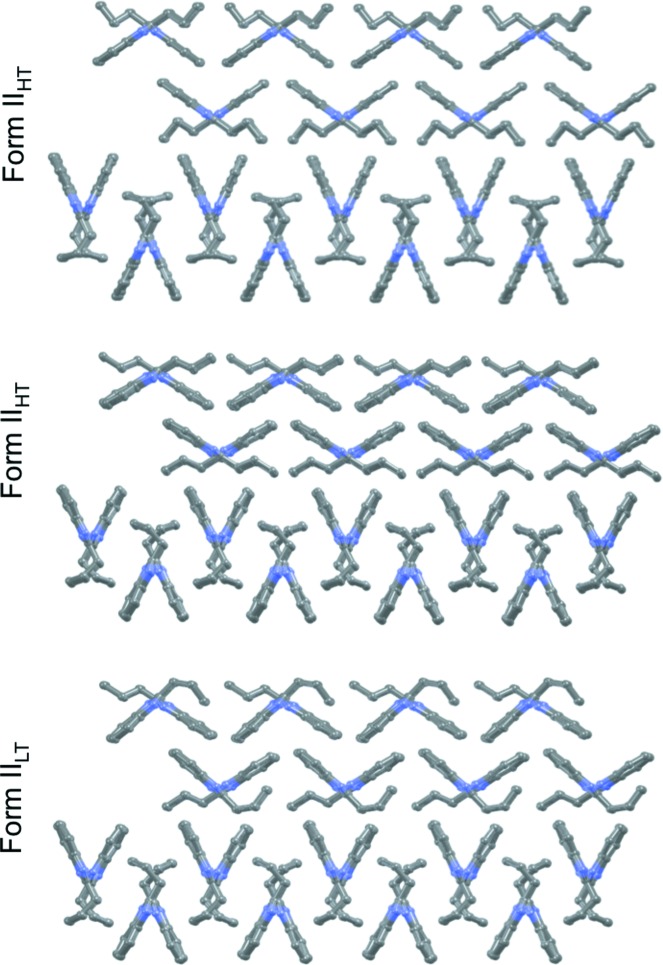
View along the hydrogen-bonded axis of form **II** phases at HT, RT and LT. Hydrogen atoms have been omitted for clarity.

Form **II**_HT_ undergoes two consecutive phase transformations from high-temperature **II**_HT_ to room-temperature **II**_RT_, and from **II**_RT_ to low-temperature (**II**_LT_). All phases are very similar; they have identical packings (Fig. 12 ▸) and mostly differ by conformational variations. As described above, only conformational adjustments are observed in the **II**_HT_–**II**_RT_ transition, whilst a conformational change (CL→CT+) is required for **II**_RT_–**II**_LT_. These form **II** phase changes are reversible and can clearly be detected by DSC: they are endothermic on heating and exothermic on cooling. Given the structural characteristics of the phases **II**_HT_ and **II**_RT_, we can conclude that they transform *via* order–disorder phase transitions. For the low-temperature transition, the structural difference between the connected phases points to a displacive-type phase transition.

### 3.5. Discussion   

The phenomenon of polymorphism has been studied and reported for decades and its definition has not changed significantly from that proposed by McCrone (1965 ▸). All of the reported polymorphs and phases of 2PrBzIm contain the same type of primary supramolecular structure, consisting of chains of molecules bonded through N—H⋯N hydrogen bonds. The categorically different packing of the molecular chains in forms **I** and **II**_HT/RT/LT_ lead to a classification of form **I** relative to any of the form **II** modifications as different polymorphs (some of which coexist under the same pressure and temperature conditions). Since forms **I** and **II** also require a conformational change from a linear to a twisted conformer, they can also be unequivocally referred to as conformational polymorphs (Cruz-Cabeza & Bernstein, 2014 ▸). The difference in lattice enthalpy between **I** and **II** is ∼5 kJ mol^−1^, which is in line with those observed for polymorphs (Cruz-Cabeza *et al.*, 2015 ▸).

We have referred to the HT, RT and LT versions of form **II** as different temperature modifications of the same form. These temperature modifications have very similar stabilities (all within less than 1 kJ mol^−1^, see Table 2 ▸) and virtually identical crystal packings (Fig. 12 ▸). These modifications cannot coexist, so each of them is stable at a different range of temperatures. The transformations between these different temperature modifications seem to be driven by an increase in density with cooling, or a decrease in density with heating (Table 2 ▸), thus resulting in the modification with an overall lower free energy for a given temperature.

We have observed a conformational change between the form **II** RT and LT modifications. We refer to **II**_RT_ and **II**_LT_ as ‘conformational phases’ since, even though the crystal packings between the structures hardly change, there is a conformational change occurring. This is an interesting observation since conformational changes are sometimes accompanied by important crystal-packing variations (Cruz-Cabeza & Bernstein, 2014 ▸) as in, for example, forms **I** and **II**. In the case of conformational phases, however, whilst there is no change in the crystal packing, there is still a requirement for the conformation to cross a conformational energy barrier (of ∼15 kJ mol^−1^ in this case, Fig. 6 ▸). Conformational phases are different to conformational polymorphs and may arise only: (i) in flexible molecules with rotatable bonds at the periphery of the molecule – whose rotations and conformational changes result in small changes of the overall molecular shape – and/or (ii) in cases where only a small proportion of unit-cell mol­ecules experience the conformational change. Since conformational polymorphs significantly change conformations and crystal packing, their properties may also change more significantly (Cruz-Cabeza *et al.* 2015 ▸); conformational phases, however, being so similar in packing and the conformational change being small, may result in phases of very similar properties (forms **II**_RT_ and **II**_LT_ differ by 0.3 kJ mol^−1^ in enthalpy). In our case study, beyond this, conformational-polymorph transitions were found to be irreversible, whilst conformational phase transitions were fully reversible. We notice that this phenomenon of conformational phases has also been observed in the polymorphs of bicalutamide (Drebushchak *et al.*, 2009 ▸), an anti-androgen drug. Given the fact that many pharmaceutical compounds have alkyl chains, conformational phases may be common in pharmaceutical materials science.

## 4. Conclusions   

We have studied the phase behaviour of 2PrBzIm as a function of temperature. This has resulted in the discovery of three new temperature phases (forms **II**_HT_, **II**_RT_ and **II**_LT_) which are related to the previously reported form **I** by conformational polymorphism. We have studied all forms, their structures and transformations in depth with various methods and provided an analysis of the structural steps required for their transformations. The phase changes on heating are driven by an increase in entropy, whilst those on cooling are driven by an increase in enthalpy (and density). This system and its transformations may thus serve as an ideal model system for benchmarking free-energy simulations in crystals.

Most interestingly, we have established that two of the temperature phases (RT and LT) are related by conformational changes despite having almost identical crystal packing. We have referred to these as ‘conformational phases’. In the case of forms **II**_RT_ and **II**_LT_, the change in conformation has a small impact on the overall packing of the structure, partly because: (i) only a quarter of the molecules in the unit cell experience the conformational change, and (ii) the rotatable bond experiencing the change is located in the periphery of the molecule. Molecules containing a long-chain hydro­carbon fragment are known to show order–disorder transitions involving changes in the conformation of the the chains, though this is usually an effect mostly observed upon heating (Maroncelli *et al.*, 1985 ▸; Snyder *et al.*, 1981 ▸), and our system, 2PrBzIm, behaves in a similar way. In 2PrBzIm, when the conformational change did not involve a packing change (forms **II**_RT_ and **II**_LT_), the observed phase transition was found to be fully reversible (conformational phases). When the conformational change also involved a packing change (forms **I** and **II**), the phase transition was found to be irreversible (conformational polymorphs).

## Supplementary Material

Crystal structure: contains datablock(s) global, IIHT, IIRT, IILT. DOI: 10.1107/S2052252518011685/lq5016sup1.cif

Structure factors: contains datablock(s) IIHT. DOI: 10.1107/S2052252518011685/lq5016IIHTsup2.hkl

Rietveld powder data: contains datablock(s) IIHT. DOI: 10.1107/S2052252518011685/lq5016IIHTsup3.rtv

Structure factors: contains datablock(s) III. DOI: 10.1107/S2052252518011685/lq5016IIRTsup4.hkl

Structure factors: contains datablock(s) IV. DOI: 10.1107/S2052252518011685/lq5016IILTsup5.hkl

Supporting information file. DOI: 10.1107/S2052252518011685/lq5016IIsup6.cml

Additional details, figures and tables. DOI: 10.1107/S2052252518011685/lq5016sup7.pdf

CCDC references: 1845850, 1845851, 1845852

## Figures and Tables

**Table 1:** Summary of crystal data, X-ray diffraction data and structure refinement

	Form **II**_HT_	Form **II**_RT_	Form **II**_LT_
Crystal data			
Chemical formula	C_10_H_12_N_2_		
*M*_r_	160.23		
Crystal system, space group	Orthorhombic, *Pcam*	Orthorhombic, *Pca*2_1_	Monoclinic, *Pna*2_1_
Temperature (K)	380	200	150
*a*, *b*, *c* (Å)	8.9957 (1), 21.5226 (3), 9.8982 (1)	9.0006 (1), 20.7778 (1), 9.7487 (1)	8.6825 (8), 42.095 (6), 9.7523 (9)
α, β, γ (°)	90, 90, 90	90, 90, 90	90, 90, 90
*V* (Å^3^)	1916.39 (4)	1823.13 (2)	3564.3 (7)
*Z*, *Z*′	8, 2	8, 2	16, 4
*V*/*Z* (Å^3^)	239.6	227.9	222.8
Density (g cm^−3^)	1.104	1.169	1.194
μ (mm^−1^)	0.52	0.55	0.56
Specimen size (mm)	Cylinder, 10 × 0.5	0.45 × 0.20 × 0.02	0.45 × 0.20 × 0.02
			
Data collection			
Diffractometer	Stoe STADI-P	Oxford Diffraction SuperNova	Oxford Diffraction SuperNova
Radiation type (Å)	Cu *K*α_1_	Cu *K*α	Cu *K*α
Specimen mounting	Quartz capillary	Single crystal	Single crystal
Data collection mode	Debye ω scan	ω scan	ω scan
Absorption correction	None	Empirical	Empirical
No. of measured, independent and observed reflections	–	31290, 3575, 3515	65861, 13647, 12984
*R*_int_	–	0.028	0.041
θ_min_, θ_max_, θ_step_ (°)	2.50, 20.0, 0.01	4.3, 74.1	4.2, 74.1
			
Refinement			
*R* factors and goodness of fit	*R*_p_ = 0.039, *R*_wp_ = 0.051, *R*_exp_ = 0.040, *R*(*F*) = 0.044, *S* = 1.28	*R*[*F* ^2^ > 3σ(*F* ^2^)] = 0.031, *wR*(*F* ^2^) = 0.044, *S* = 3.06	*R*[*F* ^2^ > 3σ(*F* ^2^)] = 0.041, *wR*(*F* ^2^) = 0.065, *S* = 3.53
No. of reflections	3500 (points)	7015	13647
No. parameters/restraints	77/28	218/24	433/24
H-atom treatment	H-atom parameters constrained		
Δρ_max_, Δρ_min_ (e Å^−3^)	0.11, −0.11	0.22, −0.22	0.25, −0.3
Absolute structure	–	1606 Friedel pairs used in the refinement	3130 Friedel pairs used in the refinement

Computer programs: *CrysAlis PRO* (Agilent, 2014 ▸), *SIR2002* (Burla *et al.*, 2003 ▸), *JANA2006* (Petříček *et al.*, 2006 ▸), *DIAMOND* (Brandenburg, 2016 ▸), *ORTEP* (Farrugia, 2012 ▸) and *Mercury* (Macrae *et al.*, 2006 ▸).

**Table 2:** Summary of key descriptors for the polymorphs and phases of 2PrBzIm

	Form **I**	Form **II**_LT_	Form **II**_RT_	Form **II**_HT_
Space group	*P*2_1_2_1_2_1_	*Pna*2_1_	*Pca*2_1_	*Pcam*
*Z*′	4	4	2	2
*Z*	16	16	8	8
Density (g cm^−3^) (*T* in K)	1.225 (100)	1.194 (150)	1.169 (200)	1.104 (380)
Δ*H*_DSC_ (kJ mol^−1^)	0.0	4.4	4.8	5.1
Δ*E*_lattice_ (kJ mol^−1^)	0.0	4.6	5.9	5.9†
*Z*′ molecules in CL	4	2	1	1
*Z*′ molecules in CT+	0	2	1	1
*Z*′ molecules in CT−	0	0	0	0
CL: τ_1_,τ_2_ (°)	45–50‡, 168–177‡	44–47‡, 176	47, 177	40, 180
CT+: τ_1_, τ_2_ (°)	–	44–84‡, 67–75‡	82, 71	76, 85

† Converts to form **II**_RT_ during optimization.

‡ Adjustment ranges.
