# Supplementary material for: Conformational aspects of polymorphs and phases of 2-propyl-1H-benzimidazole
Source: IUCrJ. 2018 Sep 14;5(Pt 6):706–15. doi: 10.1107/S2052252518011685 (PMC6211527; doi:10.1107/S2052252518011685)
Supplement: Supplementary file 7 [file m-05-00706-sup7.pdf]

IUCrJ

Volume 5 (2018)

Supporting information for article:

Conformational aspects of polymorphs and phases of 2-propyl-1*H*-benzimidazole

Fco. Javier Zuñiga, Aurora J. Cruz-Cabeza, Xabier M. Aretxabaleta, Noelia de la Pinta, Tomasz Breczewski, María Mar Quesada-Moreno, Juan Ramón Avilés-Moreno, Juan Jesús López-González, Rosa M. Claramunt and Jose Elguero

Conformational aspects of polymorphs and phases of 2-propyl-1*H*-benzimidazole

Contents

1. **Crystal preparation by sublimation**
   - **Fig S1.** Experimental device for preparation of crystals by sublimation
   - **Fig S2.** Microscopic images of crystal grown by sublimation
2. **DSC. Thermal behaviour**
   - **Fig S3.** DSC runs of the first cooling and heating cycles
   - **Fig S4.** DSC curves showing the evolution of the low temperature transition peak in successive cooling and heating cycles.
   - **Fig S5.** Flow heat in a first heating-cycle around the **I-II$_{HT}$** transitions
   - **Fig S6.** DSC curve of a heating cycle between forms **II$_{RT}$** and **II$_{HT}$**
   - **Fig S7.** DSC curve of a heating cycle between forms **II$_{LT}$** and **II$_{RT}$**
3. **IR and VCD measurements**
   - **Fig. 8.** IR and VCD spectra of **2PrBzIm** compound at different temperatures
4. **X-ray powder diffraction**
   - **Fig S9.** Diffraction patterns recorded in the temperature range 333-398 K in (a) heating and (b) cooling cycles
   - **Fig S10.** Superposition of powder difractogramas of form **I** at room temperature (blue) and form **II$_{HT}$** at 378 K(red)
   - **Fig S11.** Superposition of powder difractogramas of phases **II$_{RT}$** at room temperature (blue) and **II$_{HT}$** at 378 K (red)
5. **Structural refinements**
   - **Fig S12.** Rietveld refinement (JANA 2006) of the X-ray powder diffraction pattern of form **II$_{HT}$** at 378 K
   - **Fig S13.** Ortep plots of molecules in the asymmetric unit in forms **II$_{RT}$** and **II$_{LT}$**
   - **Fig S14.** Schematic representation of the packing in the unit cell of polymorph **II$_{RT}$** (a) polymorph **II$_{LT}$** (b)
   - **Fig S15.** Projections along the [010]-direction showing the N-H..N′ bonds scheme in the (a) polymorph **II$_{RT}$** (b) polymorph **II$_{LT}$**
6. **Computational**
   - **Table S1.** Summary of conformation torsion angles of molecules labeled as “Mol n” in Fig. S11
   - **Table S2.** Coordinates (τ_1, τ_2) of the minima in the PES
   - **Figure S16.** Perspective view of the PES

S1. Crystal preparation by sublimation

Crystal of forms **I** and **II** (HT and RT) prepared by sublimation heating at 400 K a sample of purified commercial sample. Experimental device and obtained crystals are shown in Figs S1 and S2.

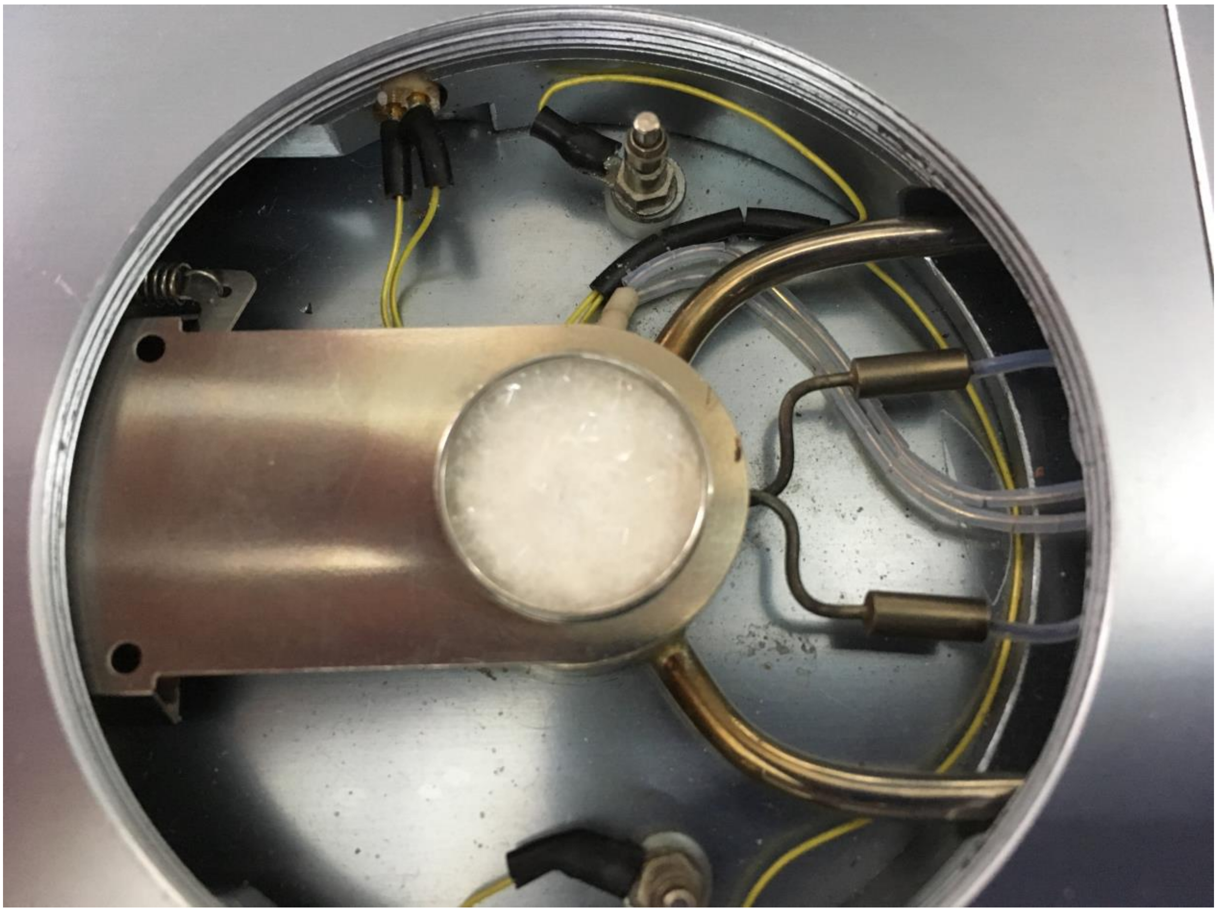

Figure S1 Hot stage used to prepared crystals in forms **I** and **II** (HT and RT) obtained by sublimation from sample-*1* type crystals (inside the ring). The sublimated compound condensates on a thin glass plate (not shown) covering the ring.

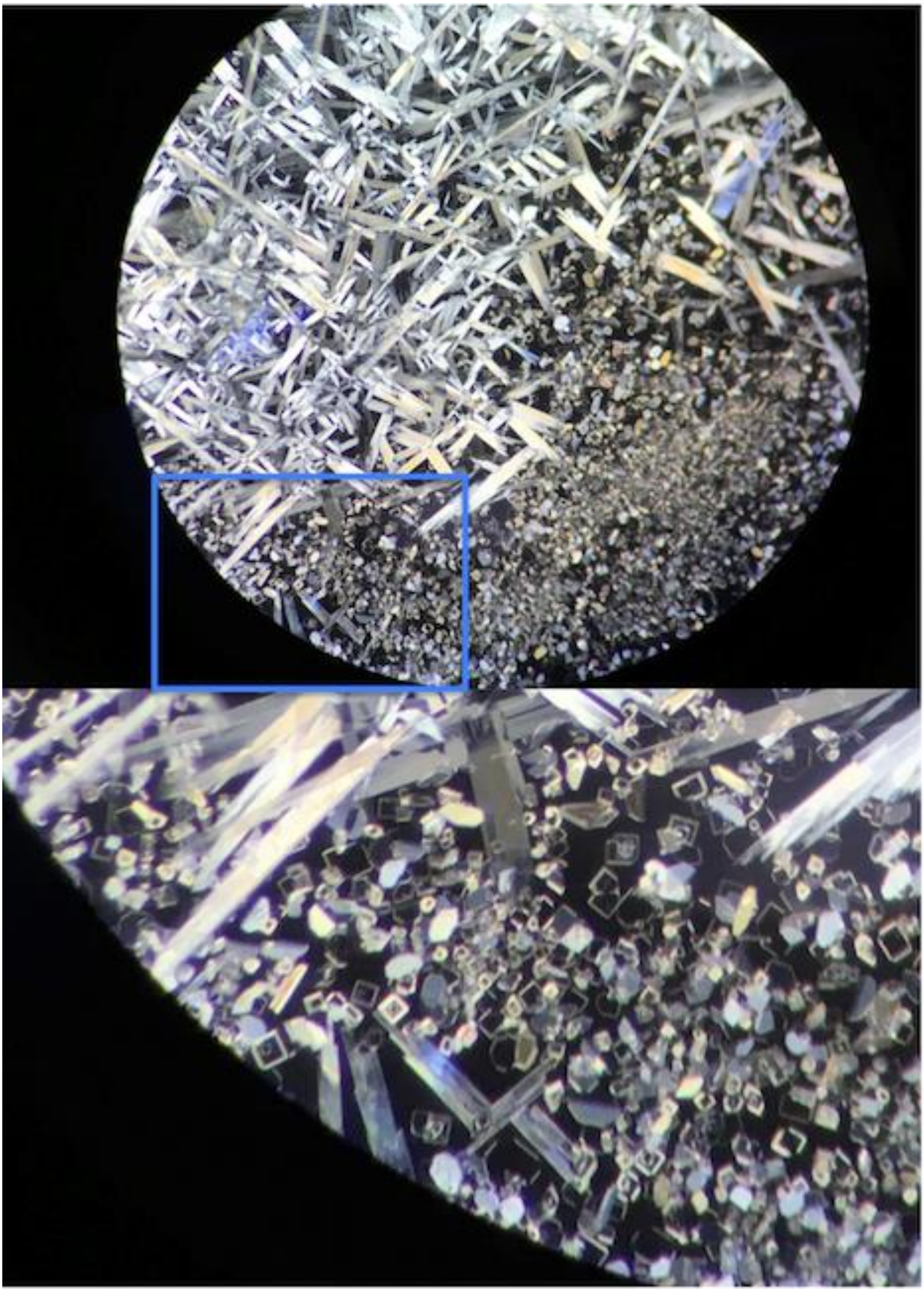

Figure S2 Microscopy image taken at room temperature showing crystal obtained by sublimation. Needle shape crystals belong to form $\mathbf{II_{RT}}$ grown on a hot area (T>384 K). The small squared shape crystals of form **I** grown on a cooler area of the glass plate on covering the heated *sample-1*.

S2. DSC. Thermal behaviour

Differential scanning calorimetric (DSC) measurements were carried out in a TA Instruments MDSC Q-2000 calorimeter in the temperature range of 140-450 K at heating and cooling rate of 10 deg/min under a helium atmosphere. Three polycrystalline samples of 2-propyl-1H-Benzomidazole of 10 up to 15 mg of mass were encapsulated in aluminum hermetic pans. The calibration of the calorimeter was performed using single-crystal sample of sapphire for calibration of specific heat values and melting point of indium for temperature and heat flow calibration. An uncertainty of 2% in ΔH was calculated from calibration with Indium.

In the first cooling run from room temperature down to 120 K, no anomaly was detected DSC. In the heating cycle, heating up to 400 K, an irreversible phase transition is observed at 381 K with entalpy of 5.1±0.3 kJ/mol (average of 4.8, 5.2 and 5.4 kJ/mol). In the next cycle cooling down 120 K, two reversible annomalies are observed at 361 K and 181 K. The entalpy of the low temperature transition increases upon cycling and reach a stable value after a few cycles. DSC runs and deteals of the transition peaks are shown in next figures.

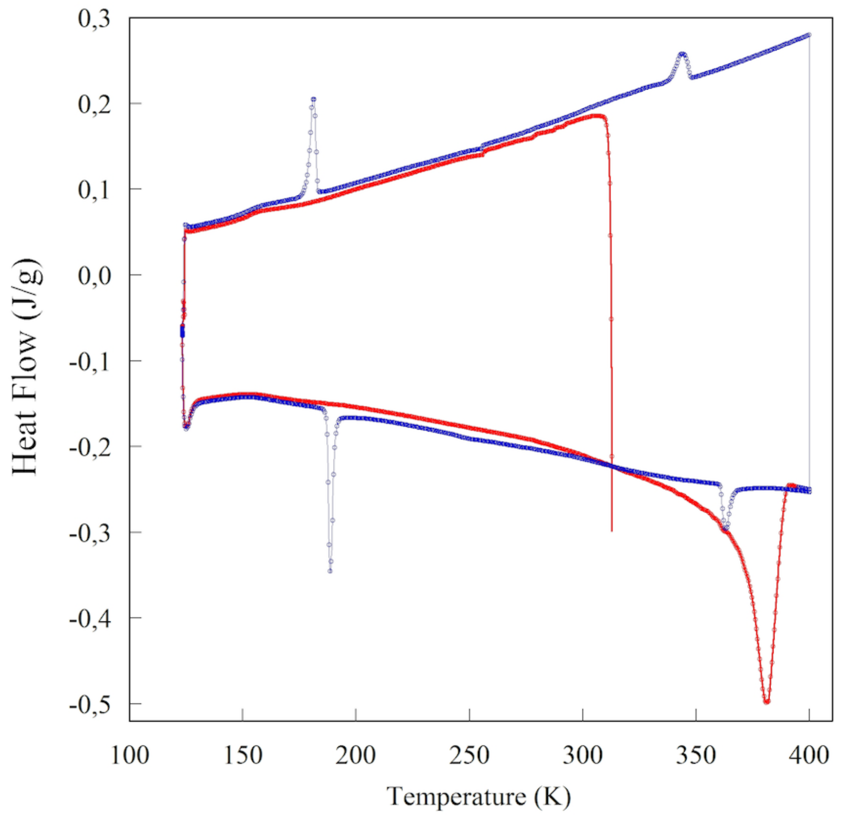

Figure S3 Flow heat v.s. temperature, positive in cooling cycles and negative in heating cycles. First cycle (red line) starts at room temperature, cool down to 120 K and heat up to 410 K. In the next cycle (blue line), cooling from 410 K down to 120 K. Next cycles are omitted for enhance clarity.

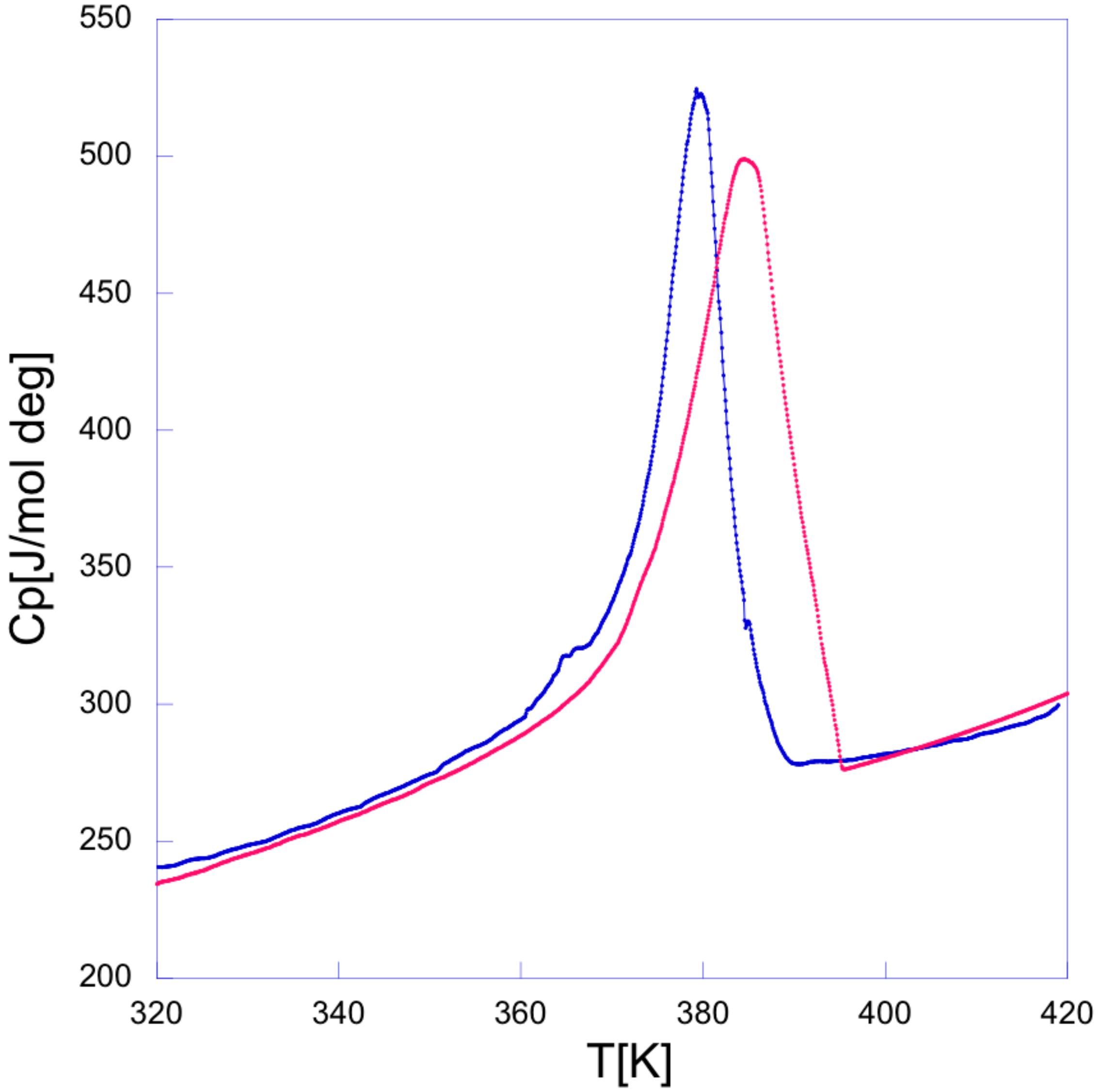

Figure S4 Flow heat in a first heating-cycle around the **I-II$_{\mathbf{HT}}$** transitions. The red curve corresponds to a recrystallized sample and the blue one to a commercial sample. The small anomaly hoisted over the blue peak corresponds to the **II$_{\mathbf{RT}}$-II$_{\mathbf{HT}}$** transition of a small fraction of form **II$_{\mathbf{RT}}$** in the commercial sample.

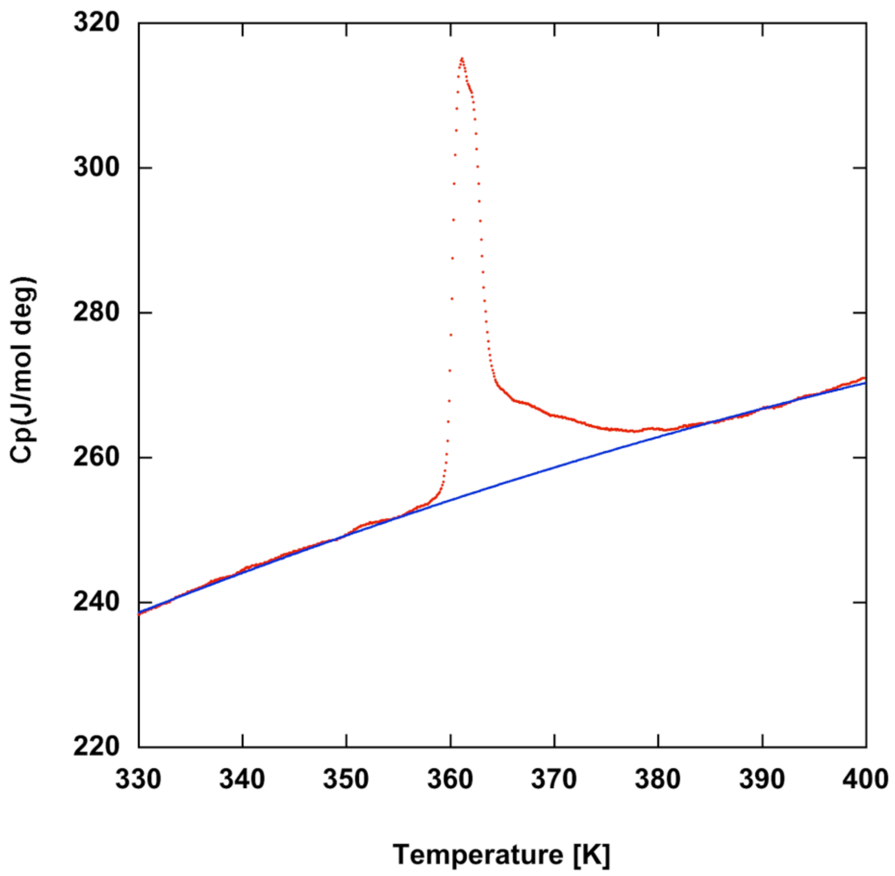

Figure S5 DSC curve of a heating cycle between forms $\mathbf{II_{RT}}$ and $\mathbf{II_{HT}}$. The blue line is the base line.

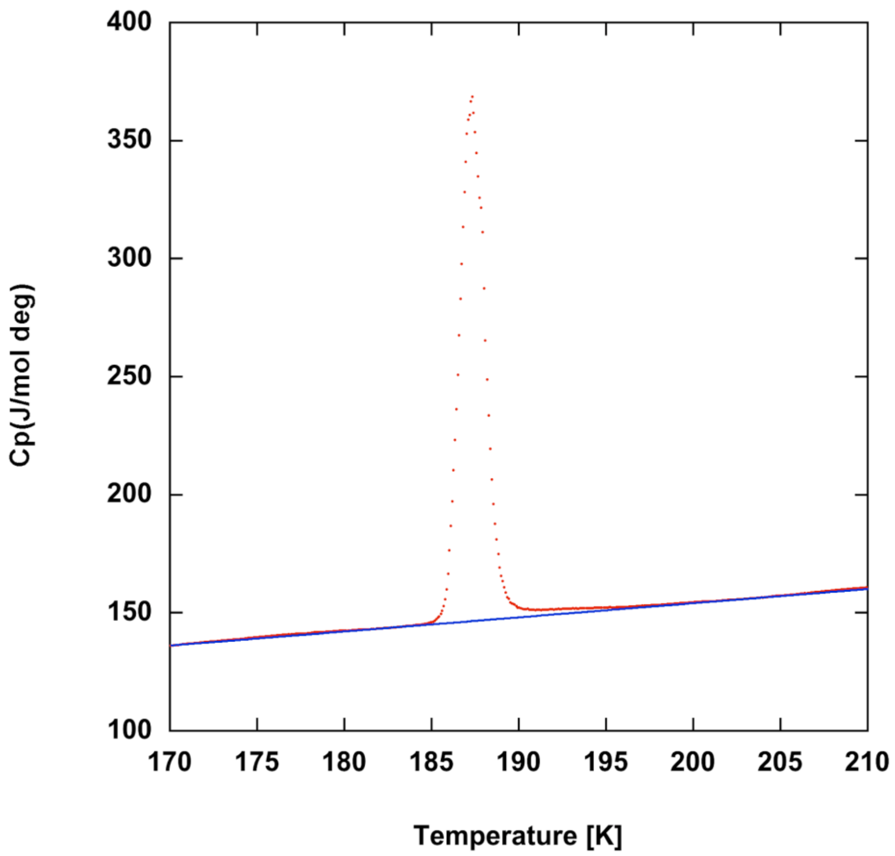

Figure S6 DSC curve of a heating cycle between forms $\mathbf{II_{LT}}$ and $\mathbf{II_{RT}}$. The blue line is the base line.

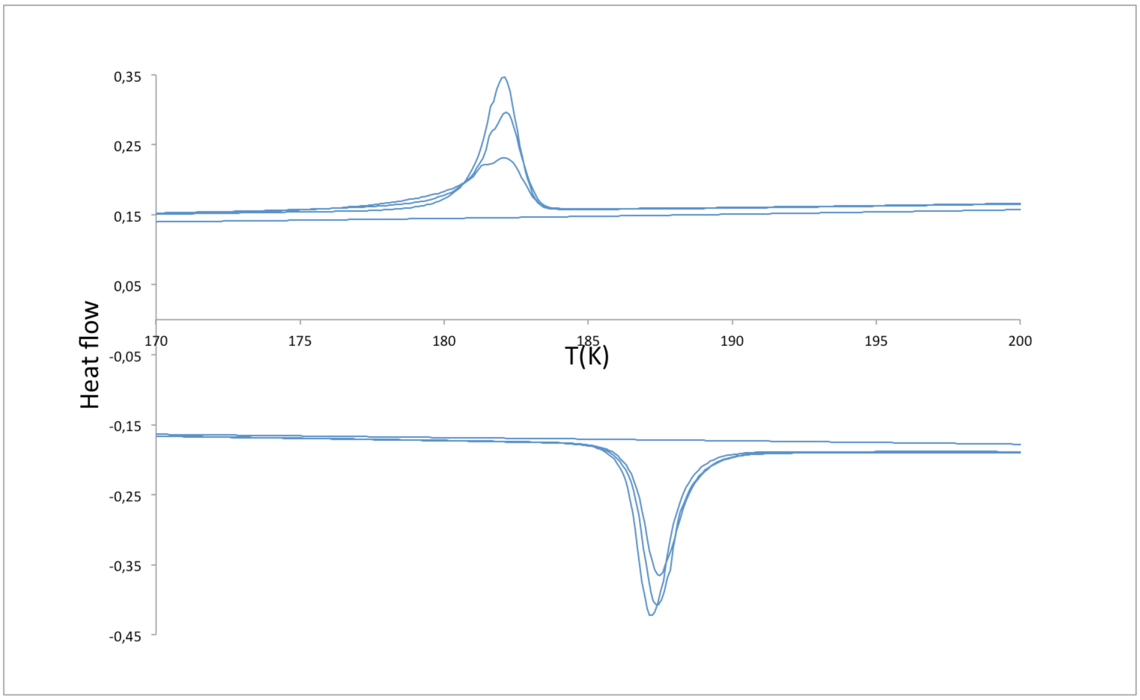

Figure S7 DSC curves showing the evolution of the low temperature transition peak in successive cooling and heating cycles. The flat line corresponds to the first cycle cooling from room temperature. Note the thermal hysteresis of 10 K.

S3. IR and VCD measurements.

For the high temperature measurements a Bruker heated sample holder (A599 model), designed for the analysis of solid samples for Raman spectroscopy, was coupled to our VCD spectrometer. Small circular BaF_2 windows (13x3 mm) were built in our laboratory starting from other bigger ones (PIKE Technologies, 32x3 mm), in order to fit them in the space available in the heated sample holder above-mentioned. Then, the holder was connected to a power supply and temperatures above 298 K for the crystalline **2PrBzIm** samples in fluorolube mineral oil could be achieved. This part of the study could not be carried out in nujol mineral oil because of the lower intensities of the spectral bands in the corresponding useful region (2000-1500 cm^{-1} and 1300-900 cm^{-1}). The VCD bands were more intense in the 1550-1420 cm^{-1} range, which can only be measured in fluorolube mull (2000-1300 cm^{-1} useful region). In this mineral oil, the VCD spectral changes according to the temperature could be followed properly.

The IR and VCD spectra were observe at temperatures below and above 378 K. The lack of chirality is evident above 378 K and when cooled again at room temperature.

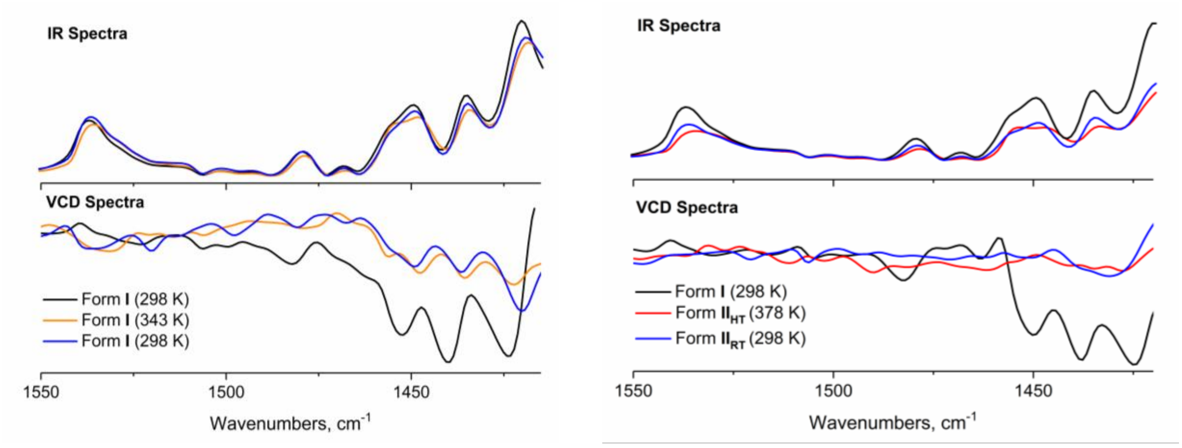

Figure S8 IR (top) and VCD (bottom) spectra of **2PrBzIm** compound in fluorolube mull at different temperatures: a) Form **I** at room temperature (298 K, black), heating until 343 K (orange) and cooling at room temperature again (blue) and b) Form **I** at room temperature (298 K, black), form $\mathbf{II}_{\mathbf{HT}}$ at 378 K (red) and form $\mathbf{II}_{\mathbf{RT}}$ at room temperature (cooling after heating, blue).

S4. X-Ray powder diffraction

X-Ray powder diffraction (XPD) spectra obtained in Debye geometry with sample in capillary of 0.5 mm. Diffractometer STOE STADIP, with Ge-monochromatized Cukα_1 radiation. Temperature monitored near the capillary with a calibrated thermocouple and controlled with a homemade heater element and Oxford Instrument ITC controller. The variation of the temperature was less that 1° during the whole exposure time.

First, diffraction patterns were recorded in heating and cooling cycles in the temperature range 333 K-398 K in steps of 5 K and heating rate of 10 K/min. Patterns are show in Fig. S9. The orthorhombic form **I** is stable up to the high temperature transition. The drastic change observed in the recorded X-ray powder diffraction diagram around 380 K led to conclude that, the first anomaly observed in DSC at 384 K correspond to a reconstructive type transition and lead to a new structure identified as orthorhombic with lattice parameter $\mathbf{a}_{\mathrm{II}}$ = 8.9957(1), $\mathbf{b}_{\mathrm{II}}$ = 21.5226(3) and $\mathbf{c}_{\mathrm{II}}$ = 9.8982(1) Å. Note that the transition temperature observed in DSC correspond the maximum of the Cp signal but transitions starts around 370 K. Cooling down to room temperature the recorded spectra does not show major changes so that the transition observed in DSC at 361 K does not involves a big structural modifications. The diffraction pattern at room temperature of polymorph $\mathbf{II}_{\mathbf{RT}}$ was indexed as orthorhombic with lattice parameters $\mathbf{a}_{\mathrm{III}}$ = 8.975(3), $\mathbf{b}_{\mathrm{III}}$ =21.134(6) and $\mathbf{c}_{\mathrm{III}}$ = 9.823(2) Å. XPD patterns of forms **I**, $\mathbf{II}_{\mathbf{HT}}$ and $\mathbf{II}_{\mathbf{RT}}$ are shown in Figs. S10 and S11. The crystallographic characterization of polymorph $\mathbf{II}_{\mathbf{LT}}$ was done by means of single crystal X-ray diffraction and is described below.

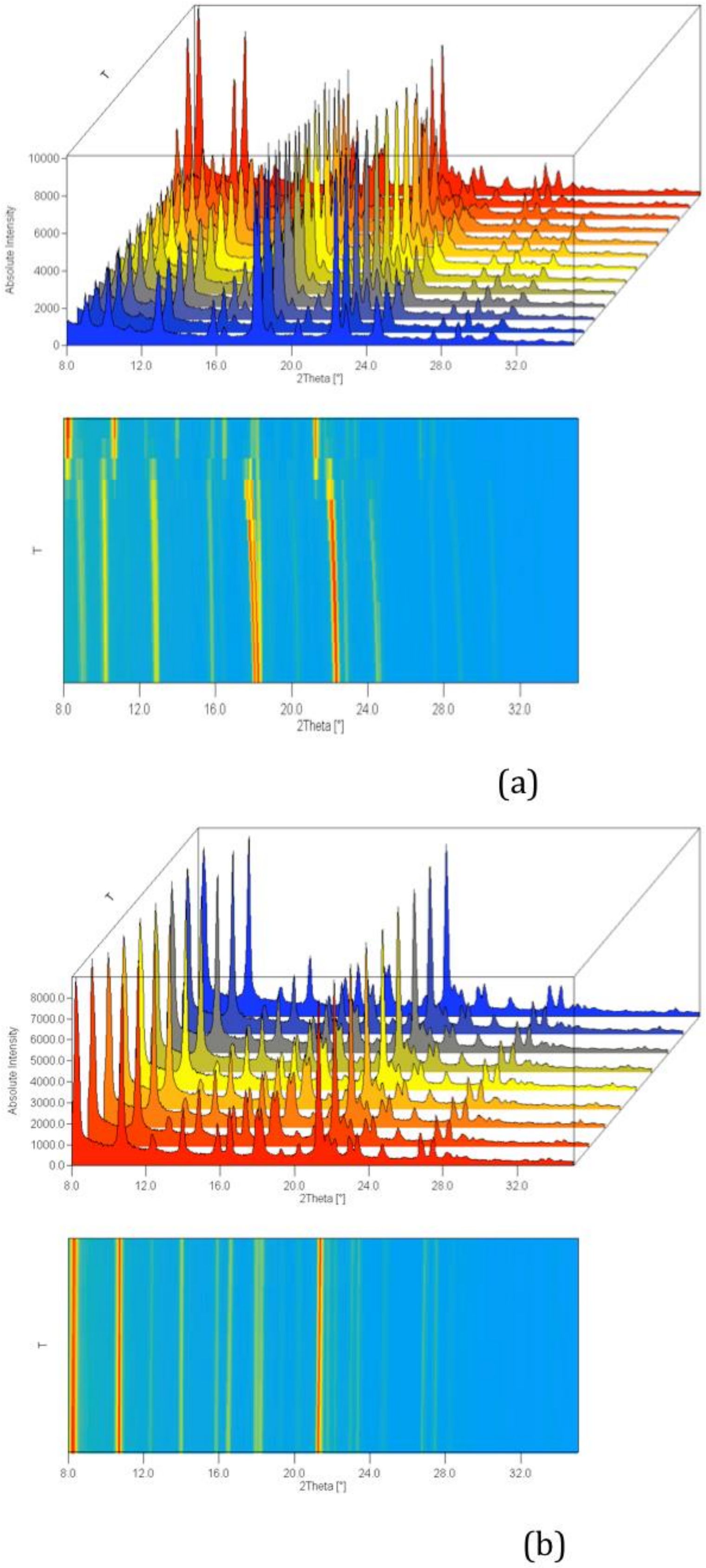

Figure S9 XRD patterns recorded in steps of 5 K in the temperatura range 333 K-398 K in (a) heating and (b) cooling cycles respectively.

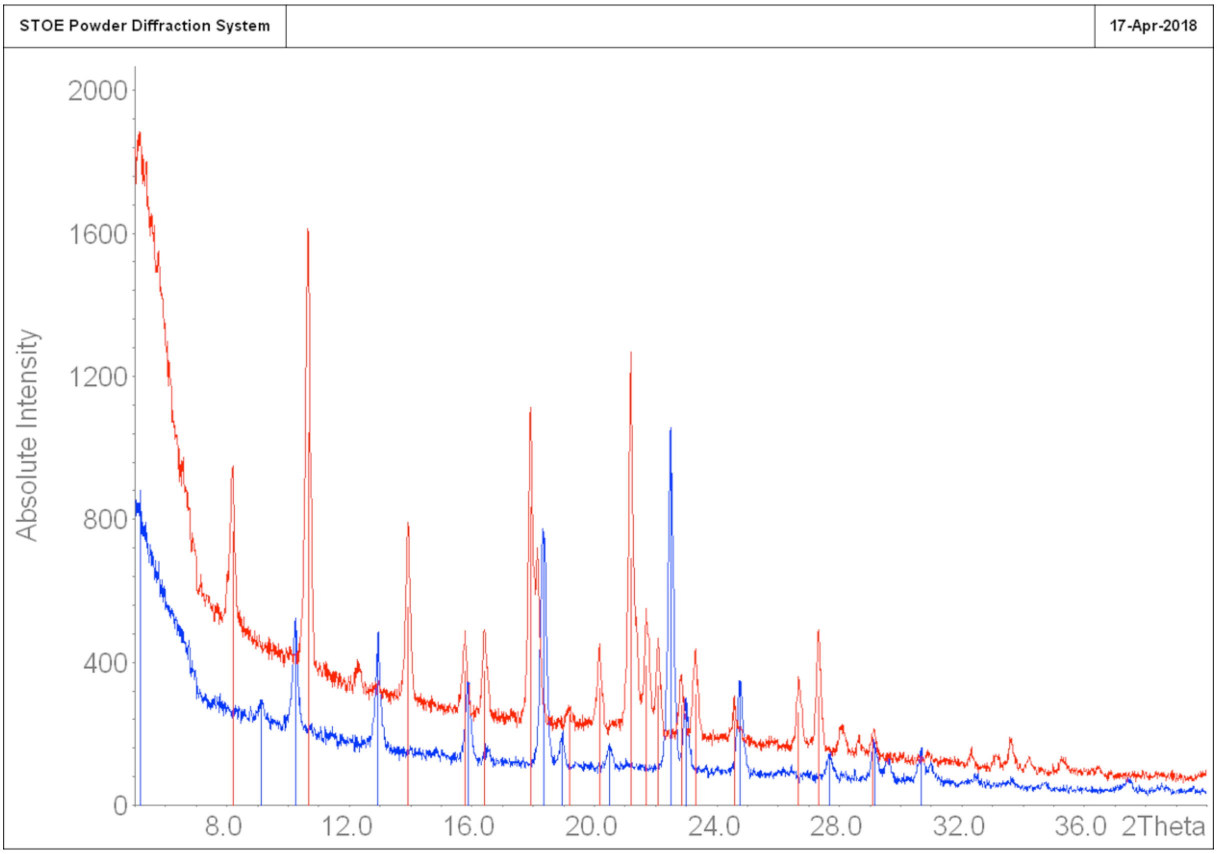

Figure S10 Superposition of powder diffractograms of form **I** at room temperature (blue) and form **II**$_{\mathbf{HT}}$ at 380 K(red).

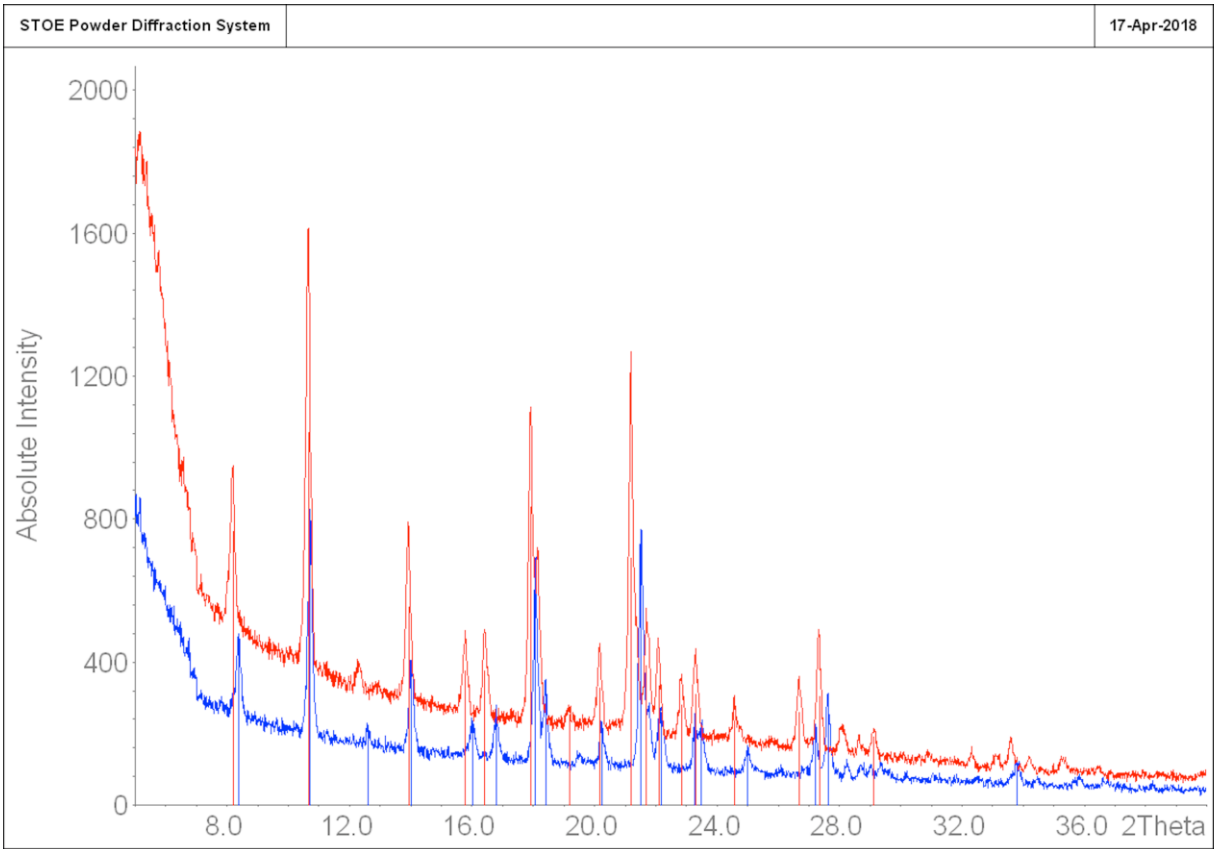

Figure S11 Superposition of powder diffractograms of phases **II$_{RT}$** at room temperature (blue) and **II$_{HT}$** at 380 K (red).

S5. Structural refinement

S5.1. Rietveld refinement of form II$_{HT}$

The structural model of **2PrBzIm** in the high temperature form **II$_{HT}$** was refined through the Rietveld method based on the XPD data measured at 380 K. The indexing peaks are compatible with extinctions of the space group *Pcam* and *Pcac*2$_1$ (Z = 8, Z' = 2). Both space groups were tested using as starting model that obtained for form **II$_{RT}$** (see below). Refinement with the non-centrosymmetric space groups becomes unstable and was discarded. In the centrosymmetric space group the molecules are disordered between equally populated positions symmetry related by the mirror plane. The refinement was performed including geometrical restrictions for planar benzimidazole and regular six carbon-atoms ring. Hydrogen atoms bonded to the imidazole fragment were assigned to the N atom with the largest intra-molecular C-N-C' angle. The H-atoms were calculated in geometrically restricted in idealized positions using riding model (C-N=0.87, Å C-H = 0.98 Å) and their isotropic displacements parameters set to 1.2Ueq of the parent N or C atom. The structure was refined using isotropic thermal displacements restricted to a unique value. Agreement between observed and calculated patterns is presented in Fig. S12. Crystal data and details of the refinement are given in Table 2.

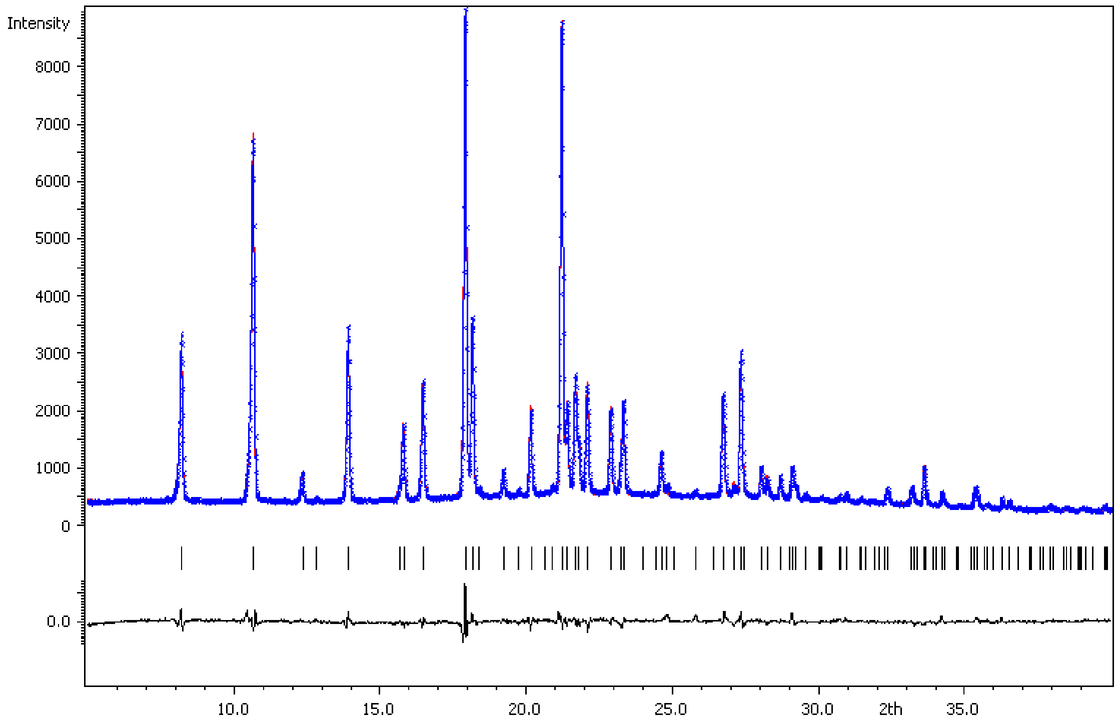

Figure S12 Rietveld refinement (JANA 2006) of the X-ray powder diffraction pattern of form $\mathbf{II}_{HT}$ at 380 K.

S5.2. Single crystal X-ray refinement of forms $\mathbf{II}_{RT}$ and $\mathbf{II}_{LT}$

Suitable single crystals in form $\mathbf{II}_{\mathbf{RT}}$ were obtained by sublimation as described in **S1**. The selected crystal used to collect intensities at 200 K (in $\mathbf{II}_{\mathbf{RT}}$**)** was also used for measurements at 150 K ($\mathbf{II}_{\mathbf{LT}}$). Experimental details are given in Table 2. The global diffraction pattern of form $\mathbf{II}_{\mathbf{RT}}$ was interpreted as orthorhombic with systematic absences compatible with the *Pcam* and $Pca2_1$ space groups (Z=8, Z´=2). The structural solution (SIR2002, Burla et al., 2003) confirms the non-centrosymmetric space group.

The diffraction pattern of polymorph $\mathbf{II}_{\mathbf{LT}}$ at 150 K shows new weak diffraction spots (superstructure reflections) indicating a doubling the period along the ***a*** axis. The global diffraction pattern was interpreted as orthorhombic with systematic absences compatible with the $Pna2_1$ space group (Z = 16, Z' = 4) confirmed with the structural refinement. The starting model in the refinement was generated as a subgroup of the parent form $\mathbf{II}_{\mathbf{RT}}$. Both structures ($\mathbf{II}_{\mathbf{RT}}$ and $\mathbf{II}_{\mathbf{LT}}$) were refined with the same type of intra-molecular restriction. In the final steps of the refinements all the individual atomic coordinates free to vary but restricting the six carbon-atoms rings to a regular shape and the benzimidazole in flat configuration. At the end of the refinement anisotropic thermal displacements were refined for all non-hydrogen atoms. The H-atoms bonded to N atoms were located in difference electron density maps and their positions, together with the rest of the H-atoms, were calculated in idealized positions using riding model (C-N=0.87, Å C-H = 0.98 Å) and their isotropic displacements parameters set to 1.2Ueq of the

parent N or C atom. Crystal data, data collection and refinement details for **II$_{RT}$** and **II$_{LT}$** are given in Table 2. Ortep plots showing the molecular configuration in forms **II$_{RT}$** and **II$_{LT}$** are shown in Fig. S13. Projections showing the molecular packing are shown in Figs. S14 and S15.

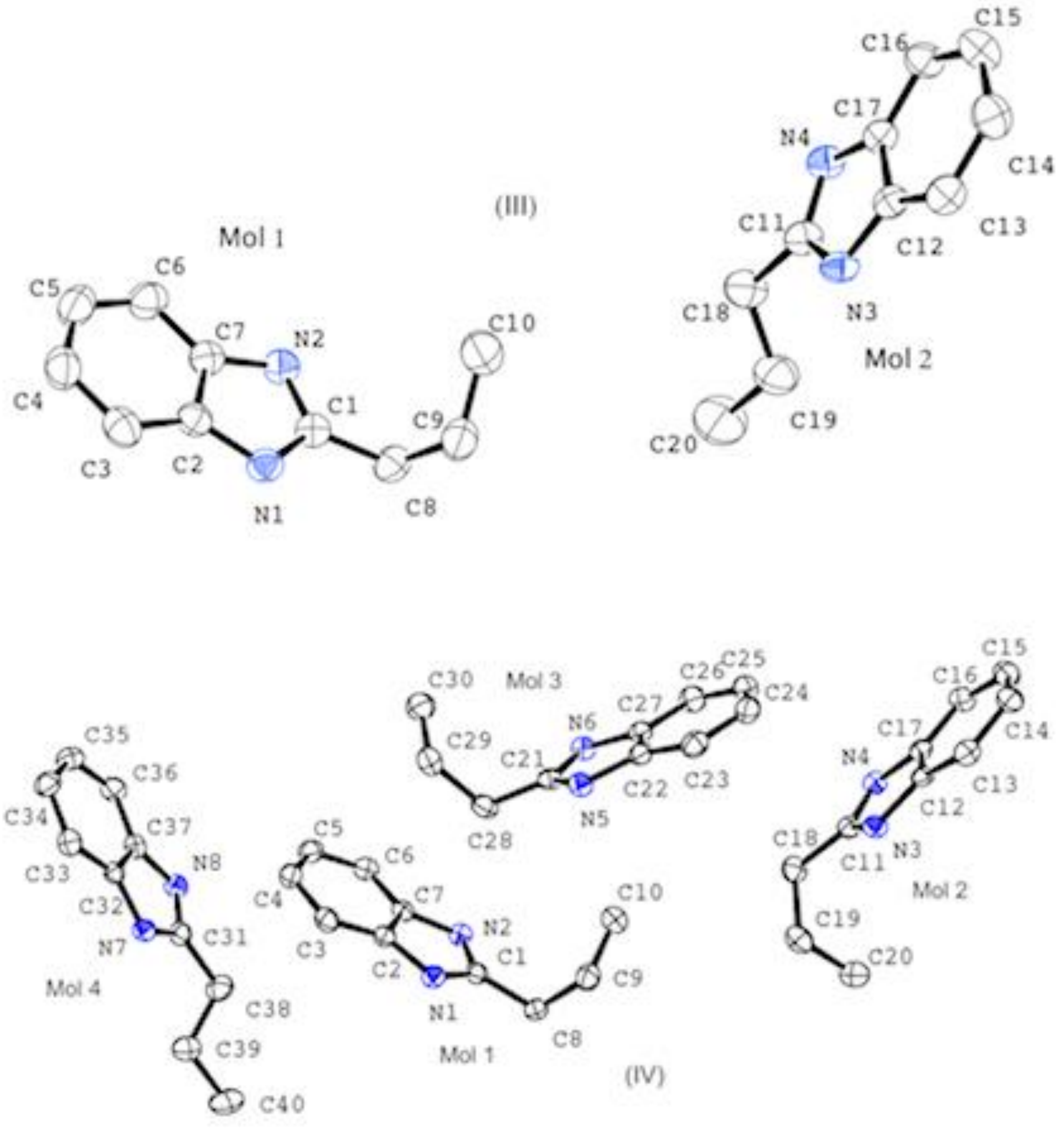

Figure S13 Ortep plots of molecules in the asymmetric unit in forms **II$_{RT}$** and **II$_{LT}$**. Configurations of molecules in polymorph **II$_{HT}$** are similar to that of **II$_{RT}$** and are not represented. The molecular labels “Mol n” are added to help identification in Table S1.

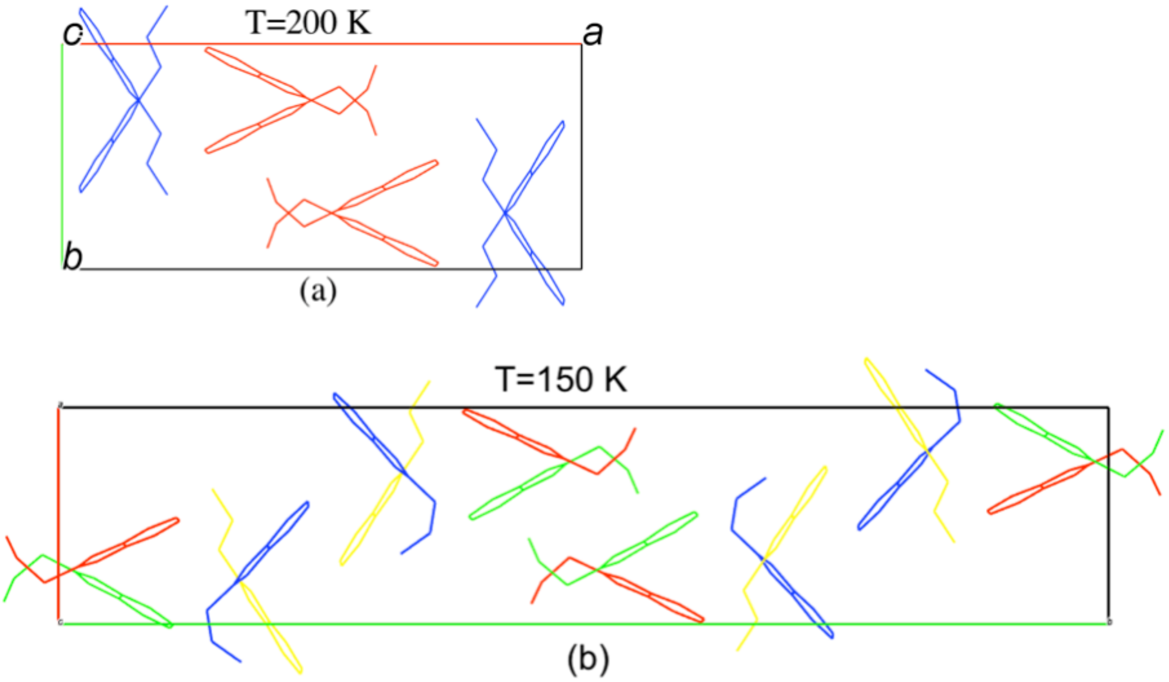

Figure S14 Schematic representation of the packing in the unit cell of polymorph **II$_{RT}$** (a) polymorph **II$_{LT}$** (b). Symmetry independent molecules are colored coded. Origins in structures **II$_{RT}$** and **II$_{LT}$** are shifted.

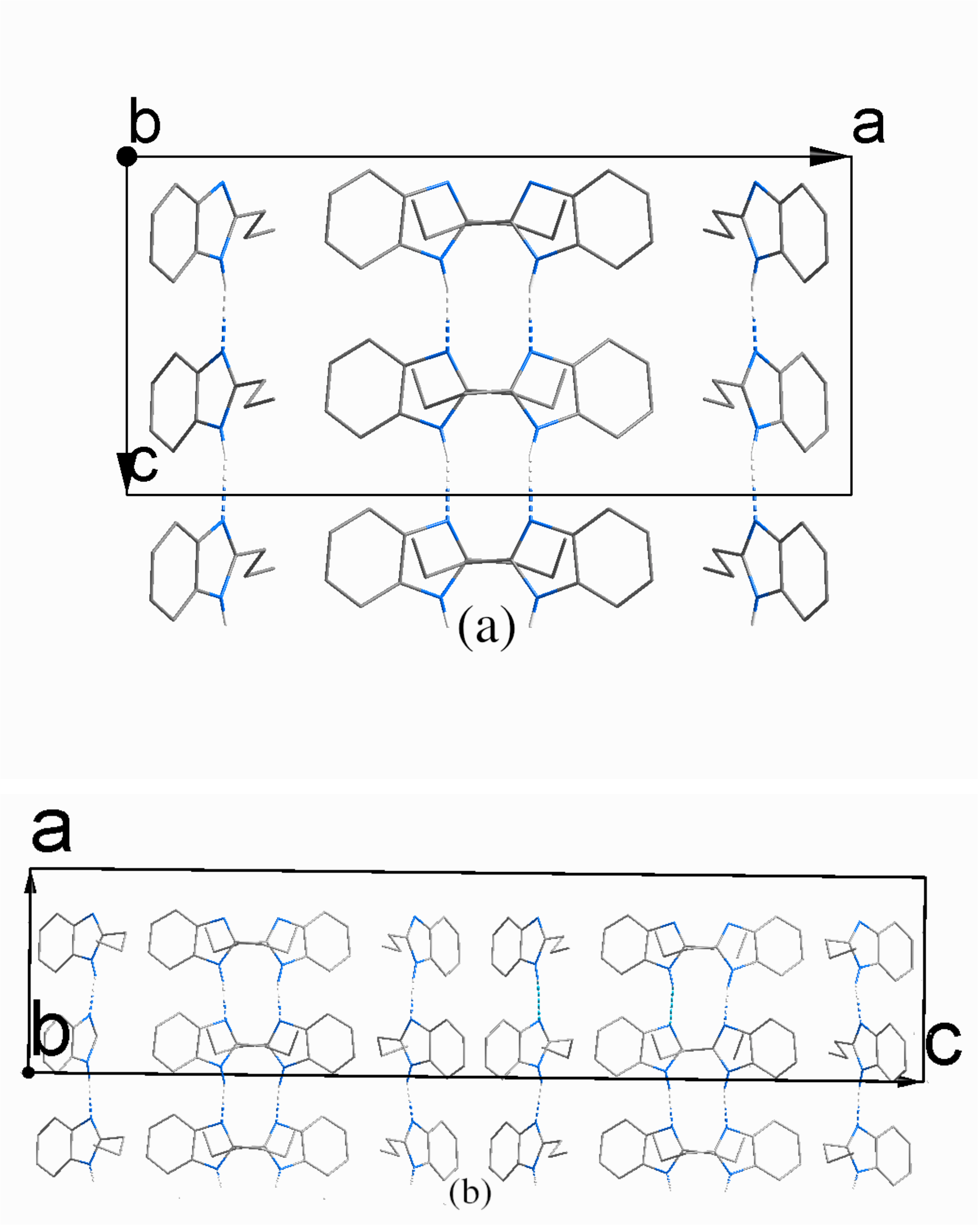

Figure S15 Projections along the [100]-direction showing the N-H..N′ bonds scheme in the (a) polymorph **II**$_{RT}$ (b) polymorph **II**$_{LT}$. H-bonds not involved in N-H…N′ bridges are omitted for enhanced clarity.

S5. Computational studies on 2-propyl-1H-benzimidazole (2PrBzIm)

Table S1 Summary of conformation torsion angles τ_1 and τ_2 of molecules labeled as "Mol n" in Fig. S11. Torsion angles values of independent molecules are typeset in black and values typeset in grey correspond to symmetry related molecules.

	**I** (295) §	**II_HT** (380)	**III_RT**(200)	**II_LT** (150)
	Torsion angle τ_1			
Mol1: N1—C1—C8—C9	45(1)	−76 (2)	-81.9(2)	−81.5(2)
Mol2: N3—C11—C18—C19	–46(1)	−39 (2)	−47.4(2)	−44.6(3)
Mol3: N5—C21—C28—C29	–50(1)	76 (4)	81.9(2)	84.2(2)
Mol4: N7—C31—C38—C39	47(1)	39 (2)	47.4(2)	47.6(3)
	Torsion angle τ_2			
Mol1: C1—C8—C9—C10	168.3(8)	−85 (2)	-71.1(2)	−72.4(3)
Mol2: C11—C18—C19—C20	177.4(8)	180 (2)	−177.4(2)	−67.1(3)
Mol3: C21—C28—C29—C30	–172.6(8)	85 (2)	71.1(2)	75.0(3)
Mol3: C31—C38—C39—C40	168.5(8)	180 (2)	-177.4(2)	176.4(2)

§ Structural data from Cabildo et al. (2015)

Table S2 Intra-molecular coordinates of the conformation molecular energies minima obtained from calculations with B97D hybrid functional with a 6-311G** Pople basis set. Energy values are given with respect to the absolute minimum.

**Conformer**	**τ_1(°)**	**τ_2 (°)**	**E (kJ/mol)**
CT-	257.0615	63.3726	0.0
CL	282.3384	181.387	+2.15829506491
CT+	288.5064	297.9062	+0.0992386565596

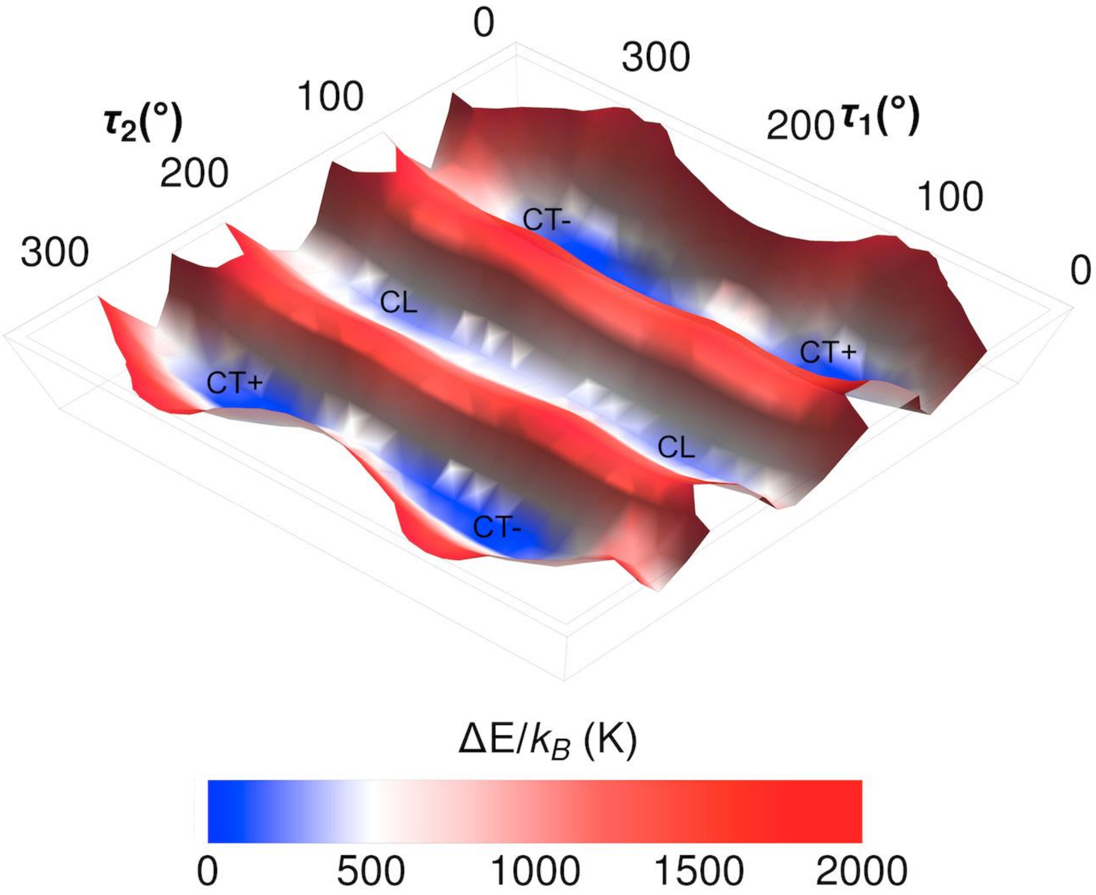

Figure S16 Perspective view of the conformational energy surface (PES) of the 2**PrBzIm** molecule calculated with B97D functional.
